# Regional and Temporal Patterns of Long-Term Pseudorabies Virus Detection and Neuropathology in the Murine CNS

**DOI:** 10.3390/pathogens15040395

**Published:** 2026-04-07

**Authors:** Viktoria Korff, Issam El-Debs, Barbara G. Klupp, Conrad M. Freuling, Jens P. Teifke, Thomas C. Mettenleiter, Julia Sehl-Ewert

**Affiliations:** 1 Department of Experimental Animal Facilities and Biorisk Management, Friedrich-Loeffler-Institut, 17493 Greifswald, Germany; viktoria.korff@fli.de (V.K.); issam.el-debs@fli.de (I.E.-D.); JensPeter.Teifke@fli.de (J.P.T.); 2 Institute of Molecular Virology and Cell Biology, Friedrich-Loeffler-Institut, 17493 Greifswald, Germany; barbara.klupp@fli.de (B.G.K.); conrad.freuling@fli.de (C.M.F.); mettenleiter@fli.de (T.C.M.)

**Keywords:** alphaherpesvirus, pseudorabies virus, mouse model, latency, in situ hybridization, brain pathology

## Abstract

Alphaherpesviruses, including Herpes Simplex Virus 1 (HSV-1) and Pseudorabies Virus (PrV), establish lifelong latency in the nervous system and can cause recurrent disease. While latency has classically been attributed to peripheral sensory ganglia, accumulating evidence indicates that the central nervous system (CNS) may also serve as a site of long-term viral persistence and reactivation. Here, we investigated the CNS as a viral reservoir using the attenuated mutant PrV-∆UL21/US3∆kin, which preferentially targets mesiotemporal brain regions. Following intranasal inoculation, mice were analyzed at 11–14, 21, 28, 42, 105, and 190 days post-infection (dpi). To assess the reactivation potential, a subset of animals received cyclophosphamide/dexamethasone at 170 dpi. Viral transcripts were detected by RNAscope™ in situ hybridization and RT-qPCR targeting the lytic gene UL19 encoding the major capsid protein and the latency-associated transcript (LAT). Histopathology included hematoxylin and eosin staining and immunohistochemistry for CD3, Iba1, GFAP, cleaved caspase-3 and viral glycoprotein gB. UL19 RNA signals displayed marked regional and temporal heterogeneity, with prominent detection in mesiotemporal structures. In contrast, LAT RNA levels remained low overall, with a transient peak during the acute phase. RT-qPCR confirmed high UL19 and LAT transcript levels during early infection, while LAT transcription returned to baseline levels thereafter. Histopathology showed a transition from acute necrotizing meningoencephalitis to prolonged low-grade inflammation with glial activation and focal apoptosis. Notably, UL19 RNA signals strongly correlated with T-cell infiltration, particularly at 42 dpi. Together, these findings define regional and temporal patterns of long-term PrV transcriptional activity and associated neuropathology in the murine CNS.

## 1. Introduction

Alphaherpesviruses such as human Herpes Simplex Virus Type 1 (HSV-1) and porcine Pseudorabies Virus (PrV) are neurotropic DNA viruses capable of establishing lifelong latency within the nervous system [1]. Following primary mucosal infection, virus particles enter peripheral sensory neurons and undergo retrograde axonal transport to peripheral and autonomic ganglia, particularly the trigeminal ganglion (TG) [2,3]. Within these neurons, the viral genome is maintained in a transcriptionally restricted state, classically referred to as latency [1,4,5,6,7,8].

Productive alphaherpesvirus infection is characterized by a temporally regulated cascade of immediate–early, early, and late gene expression [9,10]. In contrast, latency is defined by the absence of infectious virus production and highly restricted viral transcription, largely confined to latency-associated transcripts (LATs). In PrV, these include an unstable 8.4 kb transcript, the large-latency transcript (LLT), a stable intron, eleven micro-RNAs, two small noncoding RNAs, and three transcripts antisense to the major LAT [10,11,12]. LAT and their encoded microRNAs synergistically inhibit apoptosis [13] and suppress viral replication [14,15]. Establishment and maintenance of this state are thought to depend on complex interactions between the viral transcriptional programs, the host neuronal environment, and immune surveillance [16].

Reactivation from latency can be triggered by physiological or environmental stressors, leading to renewed viral transcription and replication, with spread either peripherally or towards the central nervous system (CNS) [17]. While clinically apparent reactivation typically manifests as peripheral disease [18], increasing evidence suggests that alphaherpesviruses may also establish latency within the CNS itself and exhibit intermittent transcriptional activity [19,20]. Such CNS involvement has been associated with primary or recurrent encephalitis, as well as subclinical reactivation events [21].

HSV-1 is the leading cause of herpes simplex encephalitis (HSE), a severe and often fatal condition with a predilection for mesiotemporal brain regions [22,23]. Beyond encephalitis, HSV-1 has been implicated as a potential co-factor in the pathogenesis of neurodegenerative disorders such as Alzheimer’s disease (AD) [24,25]. These observations emphasize the importance of understanding how alphaherpesviruses interact with the CNS over extended periods.

Although HSV-1 represents the most clinically relevant alphaherpesvirus in humans, PrV represents a well-established experimental model due to its close genetic and pathogenic relationship to HSV-1 [26,27]. In particular, the attenuated mutant PrV-ΔUL21/US3Δkin, lacking the functional tegument protein pUL21 and expressing a kinase-deficient pUS3, induces non-lethal encephalitis following intranasal inoculation of female CD1 mice [28]. Infected animals develop severe lymphohistiocytic meningoencephalitis predominantly affecting mesiotemporal brain regions and exhibit neurological deficits reminiscent of HSE. In contrast to wild-type PrV infection, which is uniformly lethal before substantial CNS invasion can occur, the majority of animals survive the acute phase, allowing infection to localize predominantly to the temporal lobe and enabling long-term investigation of virus–host interactions within the CNS [29].

Previous long-term studies using this model demonstrated a multiphasic disease course accompanied by persistent neuropathological alterations, including mild meningoencephalitis and gliosis, detectable for several months post-infection [29]. These findings raised important questions regarding the mechanisms underlying prolonged CNS pathology, including whether they reflect intermittent reactivation, sustained low-level viral transcriptional activity, chronic inflammation, or secondary immune-mediated processes, as suggested by experimental models and human case reports [30,31,32].

To address these questions, we conducted a comprehensive long-term analysis of PrV-ΔUL21/US3Δkin infection extending up to 105 days post-infection. We used spatially resolved molecular approaches, including RNAscope™ in situ hybridization and RT-qPCR, together with histopathological and immunohistochemical analyses. These methods allowed us to systematically assess viral transcriptional activity, immune cell infiltration, and lesion development across defined CNS regions. Given the limited number of in vivo models addressing long-term PrV persistence [33], we specifically focused on identifying regional and temporal patterns of viral transcriptional activity beyond the trigeminal ganglion, with particular emphasis on mesiotemporal structures.

Together, this study provides a detailed spatiotemporal characterization of long-term PrV transcriptional activity and associated neuropathology in the murine CNS and offers new insights into mechanisms of viral latency and CNS-directed host responses during prolonged PrV infection.

## 2. Materials and Methods

### 2.1. Virus

The attenuated PrV mutant PrV-ΔUL21/US3Δkin, derived from the PrV wild-type strain Kaplan [34], was previously described [28]. Virus stocks were propagated in rabbit kidney cells (RK13) maintained at 37 °C in minimum essential medium (MEM) supplemented with 10% fetal calf serum (FCS) (Life Technologies GmbH, Darmstadt, Germany). The RK13 cells used in this study were obtained from the cell bank of the Friedrich-Loeffler-Institut (Greifswald–Insel Riems, Germany) and correspond to the RK13 cell line (ATCC CCL-37, Manassas, VA, USA).

### 2.2. Animal Experiments

Female CD1 mice (6–8 weeks old, Charles River Laboratories, Sulzfeld, Germany) were used as the standard infection model [28]. Animals were housed in groups of up to five in conventional cages (type II L) under Biosafety Level 2 (BSL 2) conditions at the experimental animal facility of the Friedrich-Loeffler-Institut, Greifswald-Insel Riems. Housing conditions included a 12 h light/dark cycle (light intensity 60%), a temperature of 20–24 °C, and ad libitum access to a standardized diet (ssniff Ratte/Maus-Haltung, Soest, Germany) and fresh drinking water. Bedding (ssniff Spezialdiäten Abedd Espen CLASSIC), nesting material (PLEXX sizzle nest, Elst, Netherlands), and environmental enrichment (PLEXX Aspen Bricks medium, mouse smart home, mouse tunnel) were provided.

After a 7-day acclimatization period, mice were deeply anesthetized by intraperitoneal injection of 200 µL ketamine/xylazine (ketamine: 60 mg/kg; xylazine: 3 mg/kg, diluted in 0.9% NaCl). A total of 10 µL of virus suspension containing 1 × 10^4^ plaque-forming units (PFU) of PrV-∆UL21/US3∆kin was administered intranasally per nostril (5 μL per nostril). Mock-infected mice received cell culture supernatant from uninfected RK13 cells.

Study 1: Twelve groups (*n* = 7 per group) were inoculated either with PrV-ΔUL21/US3Δkin or mock solution. Animals were euthanized at 21, 42, and 105 dpi. Groups 1–6 were analyzed by RNAscope™ and histopathology; groups 7–12 were analyzed by RT-qPCR.

Study 2: As described previously [29], six groups (n = 6 per group) infected with PrV-∆UL21/US3∆kin and one group mock-treated were euthanized at 28, 35, 42, 49, 84 and 168 dpi. An additional group (n = 10), 5 animals infected with PrV-∆UL21/US3∆kin and 5 mock treated, received an immunosuppression at 170 dpi via intravenous injection of 5 mg cyclophosphamide and 0.2 mg dexamethasone in 250 µL phosphate-buffered saline (PBS). These mice were sacrificed 20 days later for histopathological and RNAScope™ analyses (Advanced Cell Diagnostics, Newark, CA, USA).

Randomization was performed prior to the experiment to assign animals to treatment groups and time points. Clinical evaluation was continuously monitored (24/7) using a standardized scoring system [28] encompassing three categories: (I) external appearance, (II) behavior and activity, and (III) body weight. Scores ranged from 0 to 3; animals reaching a score of 3 in any category or 2 across all three were euthanized to meet humane endpoint criteria. For euthanasia, animals were pre-treated with Carprofen (Rimadyl, Pfizer, 10 mg/kg, subcutaneous) analgesia, followed by deep isoflurane anesthesia. Transcardial perfusion was performed via the left ventricle with PBS and subsequently 4% paraformaldehyde (PFA), as described by Gage et al. [35]. Mice were euthanized by decapitation at the level of the first cervical vertebra.

In study 1, brains from groups 1–6 were post-fixed in 4% PFA for at least one week, and brains from groups 7–12 were sectioned into six regions (see section RT-qPCR) and stored in PBS for molecular analysis. In study 2, entire heads were fixed in 4% neutral-buffered formalin and decalcified in Formical 2000 (Decal, Tallman, NY, USA) for at least three days.

### 2.3. Histopathological Analysis

Brains from three PrV-∆UL21/US3∆kin-inoculated mice euthanized at 11–14 dpi (humane endpoint), 42 dpi, and 105 dpi (study 1), as well as heads from three infected mice euthanized at 28 dpi and from the cyclophosphamide/dexamethasone group (study 2), were processed for histopathology. Additionally, brains/heads from one mock-inoculated animal (study 1) and one mock-inoculated, immunosuppressed animal (study 2) served as controls.

Each brain/head was sectioned into six coronal levels from rostral to caudal, embedded in paraffin wax, and cut at 3 µm thick sections using a rotating microtome (Hyrax M55, Zeiss, Oberkochen, Germany). Anatomical landmarks were defined according to Rao [36], resulting in six standardized levels: olfactory bulb (OB) (L1), prefrontal cortex (L2), frontoparietal cortex and basal ganglia (L3), parietal cortex, thalamus, hypothalamus, and hippocampus (Hpc) (L4), midbrain (L5), and cerebellum (Cb)/pons (L6) (Figure 2A, L1–L6). For light microscopy, sections were mounted on Super-Frost-Plus-Slides (Carl Roth GmbH, Karlsruhe, Germany) and stained with hematoxylin and eosin (H&E). Neuropathological analysis focused on CNS inflammation, neuronal necrosis and reactive gliosis.

Slides were examined using a Zeiss Axio Scope.A1 microscope equipped with 5×, 10×, 20×, and 40× N-ACHROPLAN objectives (Carl Zeiss Microscopy GmbH, Jena, Germany). Whole-slide scans were obtained with a NanoZoomer digital slide scanner (Hamamatsu, S60, Herrsching am Ammersee, Germany).

### 2.4. Immunohistochemistry

Immunohistochemistry was performed to visualize the viral glycoprotein gB, to identify infiltrating immune cells and apoptotic processes in relation to histopathological changes. Primary antibodies used are listed in Table 1.

Paraffin-embedded tissue sections were dewaxed and rehydrated. Endogenous peroxidase activity was blocked with 3% hydrogen peroxide (Merck, Darmstadt, Germany) for 10 min. Antigen retrieval was performed in 10 mM citrate buffer (pH 6.0, without detergent) for Iba1, GFAP and Cas-3, or in 10 mM Tris-EDTA buffer (10 mM Tris base, 1 mM EDTA solution, pH 9.0) for CD3. Sections were heated for 20 min in a pressure cooker (Sichler, Buggingen, Germany, NX-3213-675) for epitope demasking. After rinsing in Tris-buffered saline (TBS), non-specific binding was blocked using normal goat serum (diluted 1:2 in TBS, 30 min). Sections were incubated overnight at 4 °C with primary antibodies diluted in TBS. The following day, slides were washed with TBS and incubated with biotinylated goat anti-rabbit IgG (Vector Laboratories BA 1000, Newark, CA, USA , 1:200) for 30 min at room temperature (RT). For PrVgB, Iba1 and Cas-3 staining, detection used the avidin-biotin-peroxidase (ABC) complex (Vectastain Elite, PK 6100, Vector Laboratories), for 30 min at RT. For CD3 and GFAP, the Polymere ImmPress^®^+ System (DAKO, MP-7451, Hamburg, Germany) was applied. Antigen–antibody complexes were visualized using AEC substrate (abcam, ab64252, Cambridge, United Kingdom), yielding red signal deposition. Slides were rinsed with deionized water, counterstained with Mayer’s Hematoxylin for 10 min, and coverslipped using Aquatex (Merck).

### 2.5. Scoring of Inflammatory Cells

Inflammatory responses were assessed at 11–14, 28, 42, 105, and 190 dpi in eight anatomically defined brain regions: OB, AI, Pir, LEnt, S1, Sp5, TG and Cb. Regions of interest (ROIs) were defined according to The Mouse Brain in Stereotaxic Coordinates [37]. Each ROI was systematically evaluated in standardized coronal brain sections with selected regions evaluated in one or more anatomically defined level: OB (L1), agranular insular cortex (AI) (L2/3), piriform cortex (Pir) (L2/3/4), lateral entorhinal cortex (LEnt) (L5), primary somatosensory cortex (S1) (L2/3/4), spinal trigeminal nucleus (Sp5) (L6), Cb (L6), TG (L3/4) (Figure S1). The total value of the number of positive cells detected by light microscopy per ROI/level was determined and subsequently classified using a semi-quantitative scoring system. For ROIs analyzed at multiple levels, the highest score per region was recorded. All evaluations were performed manually at high-power magnification (20× or 40×).

CD3^+^ T-cell infiltration was semi-quantitatively scored according to a published system (Table 2 [29]). Cas-3 was scored using the same criteria. Iba1^+^ microglia/macrophages and GFAP^+^ astrocytes were qualitatively assessed as present or absent (Table 3), based on glial activation regions with CD3^+^ infiltration.

### 2.6. In Situ Hybridization (RNAscope^TM^)

Detection of PrV transcripts was performed on formalin-fixed paraffinembedded (FFPE) sections (Figure 2A) using the RNAscope™ 2.5 HD Duplex Reagent Kit (ACD Inc., Cat. No. 322430) [38].

A C1 probe targeting the lytic viral transcript UL19 (V-SHSV-UL19, 66973–68498 base pairs; ACD Inc., Cat. No. 548251) was detected as green punctate signals via horseradish peroxidase (HRP)-mediated chromogenic reaction.

A C2 probe targeting the LLT region of the LAT (V-SuHV1-LLT-O2-C2, 94664-95952 base pairs, ACD Inc., Cat. No. 1808611) was detected as red punctate signals by alkaline phosphatase (AP)-mediated chromogenic reaction (Figure 2B). Probes were mixed at a dilution of 1:50 (C2 in C1).

As technical control, the murine housekeeping gene Ppib (Mm-Ppib, Advanced Cell Diagnostics, Cat. No. 313911, C1), encoding the peptidyl-prolyl cis-trans isomerase B, was combined with ubiquitin C (Mm-Ubc, ACD. Inc., Cat. No. 310779, C2) at a dilution of 1:50 (C2 in C1). An Escherichia coli DapB probe (Duplex Negative Control Probe, Advanced Cell Diagnostics, Cat. No. 320759) served as a negative control (Figure S2).

Pre-treatment and hybridization: Slides were baked at 60 °C for 1 h, deparaffinized in xylene (2 × 5 min), and rehydrated in 100% ethanol (2 × 1 min) at RT. After air-drying, endogenous peroxidase activity was quenched using hydrogen peroxide (H_2_O_2_; Advanced Cell Diagnostics, Cat. No. 322330) for 10 min at RT. Target retrieval was performed by boiling slides in antigen retrieval buffer (Advanced Cell Diagnostics, Cat. No. 322000) using a pressure cooker (Sichler, Germany, NX-3213-675) for 15 min. Slides were washed in distilled water, and dehydrated in 100% ethanol. After air-drying, sections were circumscribed with a hydrophobic barrier pen (Vector Laboratories, H-4000) to confine reagent application to the tissue area. Slides were then treated with Protease Plus (Advanced Cell Diagnostics, Cat. No. 322330) for 15 min at 40 °C in the Advanced Cell Diagnostics HybEZ™ II Hybridization System (Advanced Cell Diagnostics, Cat. No. 321711) (HybEZ oven). Slides were rinsed in distilled water and incubated with the specific probes and controls for 2 h at 40 °C in the HybEZ oven, followed by two washes (2 min each) in the wash buffer (Advanced Cell Diagnostics, Cat. No. 310091). Afterwards, the slides were stored overnight at RT in a 5× saline–sodium citrate buffer (SSC) (20×X SSC, 1:4, diluted in distilled water; 20x SSC: 175 g of NaCl and 88 g of sodium citrate in 1 L distilled water, pH = 7.0).

Signal amplification and detection: On the following day, slides were washed twice in wash buffer, and preamplification was performed by applying amplicon (Amp) 1 for 30 min at 40 °C. Further amplification steps were carried out sequentially with Amp 2 (15 min) and Amp 3 (30 min), both at 40 °C. Detection of C2 (LAT) was achieved with Amp 4 (15 min at 40 °C) in the HybEZ oven, followed by Amp 5 (30 min at RT), and the AP blocker Amp 6 (15 min at RT). For visualization of the red signal, slides were treated with a 1:60 mix of Red-B and Red-A (10 min at RT). Detection of the C1 (UL19) signal was performed using Amp 7–10 (Amp 7: 15 min, 40 °C; Amp 8: 30 min, 40 °C: HRP enzyme solution; Amp 9: 30 min, RT; Amp 10: 15 min, RT), followed by green chromogenic development using a 1:50 mix of Green-B to Green-A (1:50) (10 min at RT). Between each step (Amp: 1–10 and chromogen development), slides were washed twice in wash buffer for 2 min. Slides were counterstained with Mayer’s Hematoxylin (30 s at RT), blued in tap water (5 min), dried at 60 °C (15–30 min) on a heat plate (MEDITE, BD00575, Burgdorf, Germany) and cooled for 5 min. Finally, slides were immersed in fresh xylene (5 min) and coverslipped using EcoMount (Biocare Medical, BRR897L, Pacheco, CA, USA).

### 2.7. Semi-Quantitative Analysis of In Situ Hybridization

PrV mRNA expression was assessed at 11–14, 28, 42, 105, and 190 dpi in eight anatomically defined brain regions: OB, AI, Pir, LEnt, S1, Sp5, TG and Cb. ROIs were defined according to The Mouse Brain in Stereotaxic Coordinates [37]. Each ROI was systematically evaluated in standardized coronal brain sections with selected regions evaluated in one or more anatomically defined level: OB (L1), AI (L2/3), Pir (L2/3/4), LEnt (L5), S1 (L2/3/4), Sp5 (L6), Cb (L6), TG (L3/4) (Figure S1). The total value of positive signals detected by light microscopy per ROI/level was determined and subsequently classified using a semi-quantitative scoring system. For ROIs analyzed at multiple levels, the highest score per region was recorded. All evaluations were performed manually at high-power magnification (20× or 40×).

PrV mRNA expression was semi-quantitatively scored. Each RNA transcript appeared as a discrete chromogenic dot within the tissue section (LAT: red; Ul19: green). Signals were assessed separately for UL19 and LAT in each predefined brain region. Scoring was carried out as illustrated in Figure 4I, based on the number and distribution of RNA-positive signals within the ROI. Briefly, punctate signals were scored on a scale from 0 to 3: score 0 represented no detectable signal, score 1 corresponded to 1–20 signals, score 2 to 20–100 signals, and score 3 more than 100 signals. Clustered signals were evaluated separately using scores 4 and 5, with score 4 assigned to 1–20 clusters and score 5 to more than 20 clusters. All evaluations were performed at high power (20× or 40×).

### 2.8. RT-qPCR

Brains from six PrV-∆UL21/US3∆kin- or mock-inoculated mice per time point (9–10, 21, 42, and 105 dpi, study 1) were dissected into six regions: OB, Pir, TL, Cb, BS and TG. Tissues were homogenized in PBS with 5 mm steel beads (Fabrikat Martin) using a bead beater (Retsch, MM200, Haan, Germany). Total RNA was extracted using the QIAamp Viral RNA Mini Kit (Qiagen, Cat. No. 52904, Hilden, Germany) according to the manufacturer’s instructions, and RT-qPCR was performed to detect transcripts of UL19 (lytic gene) and the LAT. Custom-designed primers (Eurofins, Hamburg, Germany) were used for LAT, and validated primer-probe sets for UL19 were used [39]. Primer sequences are listed in Table 4. β-actin was used as an internal control to verify RNA integrity and successful reverse transcription. Ct values for β-actin were used to define a detection range within which viral transcripts were considered reliably detectable; samples outside this range were not subjected to quantitative normalization. A synthetic long LAT oligomer was used both as a positive control and as a standard for generation of the calibration curve. The threshold cycle (Ct) cutoff was set at 40, based on dilution series of the LAT standard. Reactions above this threshold were considered negative.

A 200 µL primer mix was prepared, containing 100 pmol each of forward and reverse primer, 2.5 µL of the fluorophore-labeled probe, and 157.5 µL of 0.1X TE buffer (pH 8.0). Each 20 μL PCR reaction consisted of 10 μL of ready-to-use mix (2x SensiFast Probe No-ROX One-Step mix), 0.2 reverse transcriptase, 0.4 RiboSafe RNase inhibitor, 0.6 µL of RNase-free water, 1.6 µL of each primer mix and 4 µL of RNA template. Amplification was performed using a Bio-Rad CFX96 thermal cycler under the following conditions: reverse transcriptase treatment at 45 °C for 15 min, inactivation of the transcriptase at 95 °C for 2 min, template denaturation at 95 °C for 15 s, and annealing/amplification of the target cDNA at 60 °C for 40 s (40 cycles). Fluorescence was measured during the elongation phase.

### 2.9. Statistical Analysis

Statistical analyses and graphical presentation were performed in GraphPad Prism 10.2.1 (GraphPad Software, Boston, MA, USA).

Ct values obtained from RT-qPCR were analyzed by two-way analysis of variance (ANOVA) with Sidak’s multiple comparison test. Geometric mean values of UL19 and LAT transcripts were compared between brain regions. Correlations between CD3^+^ T-cell scores and UL19 RNA expression in corresponding brain regions were assessed using Spearman’s rank correlation coefficient.

*p* ≤ 0.05 was considered statistically significant, and is indicated by an asterisk in the figures.

## 3. Results

Female CD1 mice (6–8 weeks old) were intranasally inoculated and sacrificed at 21, 42 and 105 dpi for RT-qPCR, RNA in situ hybridization, and histopathological analyses (study 1). Archived brain sections from a previous long-term study (study 2) using the same model [29] were reanalyzed to investigate the post-acute phase at 28 dpi. In that study, a subset of animals received cyclophosphamide/dexamethasone at 170 dpi and were sacrificed 20 days later. These tissues were included for RNA in situ hybridization and histopathology. An overview of the experimental design is shown in Figure S3.

### 3.1. Clinical Evaluation

As previously reported in mice from study 2 (0–168 dpi) [29], infection with PrV-∆UL21/US3∆kin in study 1 followed a multiphasic disease course. From day 5 post-infection, mice developed mild clinical signs including ruffled fur and reduced activity. Disease symptoms peaked between 10 and 15 dpi, with incidence rates approaching 90%. During this acute phase, mice displayed alopecic skin erosions, nasal bridge edema, mild pruritus, and behavioral abnormalities such as hyperactivity and “star gazing”. Approximately 10% of animals reached humane endpoint criteria due to severe manifestations including seizures and hyperexcitability.

**Figure 1:**
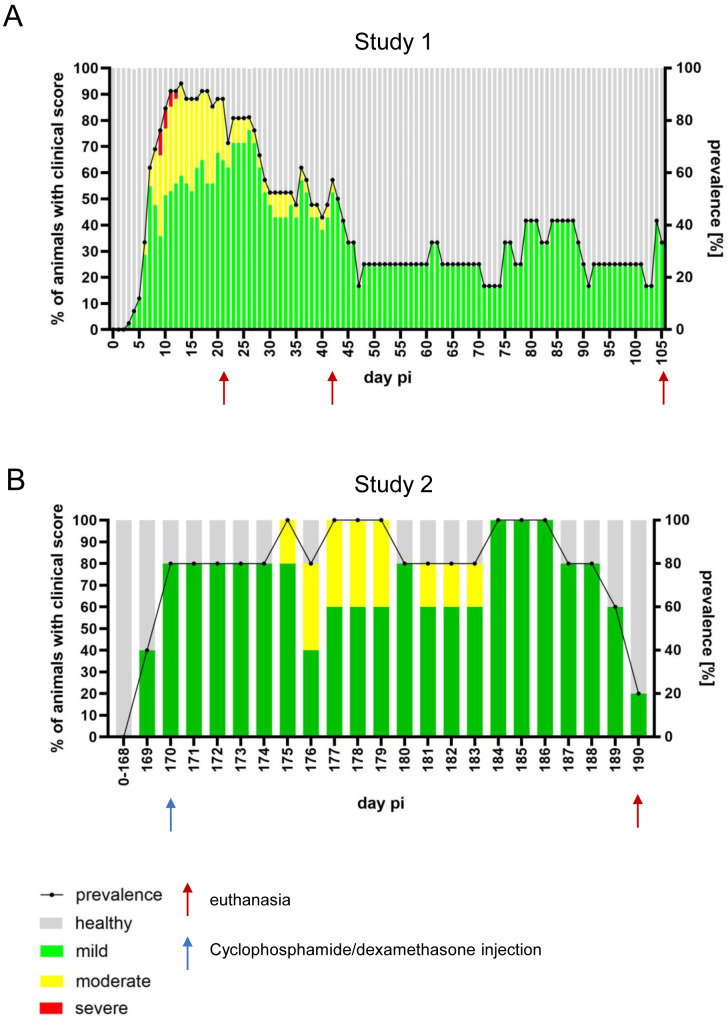
Longitudinal assessment of disease severity and incidence in PrV-∆UL21/US3∆kin-infected mice. The proportion of clinically affected animals is shown as a percentage of the total number of infected mice. Disease severity is color-coded as follows: healthy (grey), mildly affected (green), moderately affected (yellow), and severely affected (red). Percentages are plotted on the left y-axis, while the overall incidence of clinical signs is indicated by a dotted line and shown on the right y-axis. Red arrows indicate necropsy time points. (**A**) Study 1: PrV-∆UL21/US3∆kin or mock-inoculated mice (n = 7) were sacrificed at 21, 42, and 105 dpi. (**B**) Study 2: Mice (n = 5) were inoculated, monitored, and evaluated as in study 1, but received immunosuppressive treatment with cyclophosphamide/dexamethasone at 170 dpi (blue arrow), and were euthanized at 190 dpi.

By 30 dpi, predominantly mild clinical signs persisted in ~50% of mice. Between 45 and 105 dpi, incidence declined further to 20–30%, with fluctuating low-grade signs such as ruffled fur and subtle behavioral alterations (Figure 1A).

Following cyclophosphamide/dexamethasone treatment at 170 dpi in study 2, approx. 80% of animals developed mild clinical signs (ruffled fur, reduced activity) within the first 5 days post-treatment (dpt). Between 5 and 10 dpt, the disease severity increased, and all animals exhibited signs of reactivation. Approximately 50% developed moderate symptoms including “star gazing,” pruritus, and mild hunching. By 20 dpt, both incidence and severity steadily declined, with approx. 10% of animals still symptomatic at the end of the observation period (Figure 1B).

### 3.2. Spatiotemporal Detection of Lytic and Latency-Associated Transcripts

**Figure 2:**
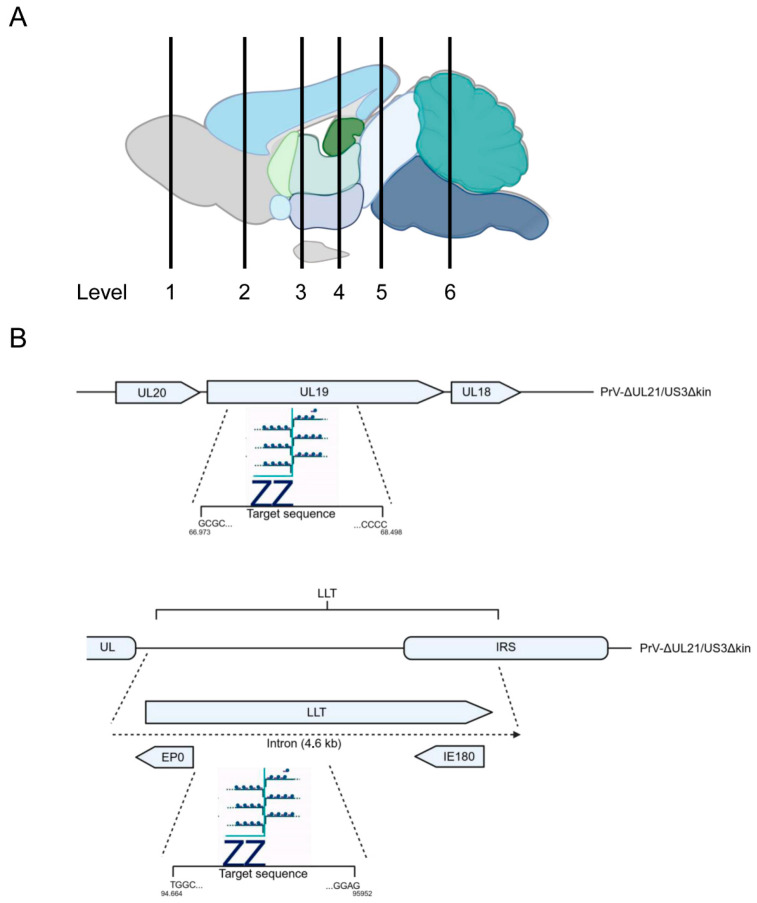
Experimental setup for RNAscope™ analysis. (**A**) Schematic overview of analyzed brain regions. Serial 3 µm coronal sections were collected for detailed analysis at six defined anatomical levels: L1 (OB), L2 (prefrontal cortex), L3 (frontoparietal cortex, basal ganglia), L4 (parietal cortex, thalamus, hypothalamus, Hpc), L5 (midbrain), and L6 (Cb/pons). (**B**) Design of UL19 (V-SHSV-UL19-C1) and LLT (V-SuHV1-LLT-O2-C2) probes used for detection of lytic and latency-associated viral mRNA. V-SHSV-UL19-C1 targets the UL19 and V-SuHV1-LLT-O2-C2 the LAT region, which is represented by the blue dots and connecting lines. Location of the probes corresponds to nucleotide positions: 66973–68498 base pairs (UL19), 94664-95952 base pairs (LLT). Created with BioRender.com.

To characterize regional and temporal viral transcriptional activity, brains from mice sacrificed at 11–14 dpi (reached humane endpoint), 28, 42, 105, and 190 dpi were analyzed. In situ RNA hybridization was performed on six standardized coronal brain sections per animal (Figure 2A, L1–L6), using the RNAscope™ method [38] with probes targeting the lytic gene UL19 and the latency-associated transcript LAT (Figure 2B).

### 3.3. CNS Sites of LAT Detection

**Figure 3:**
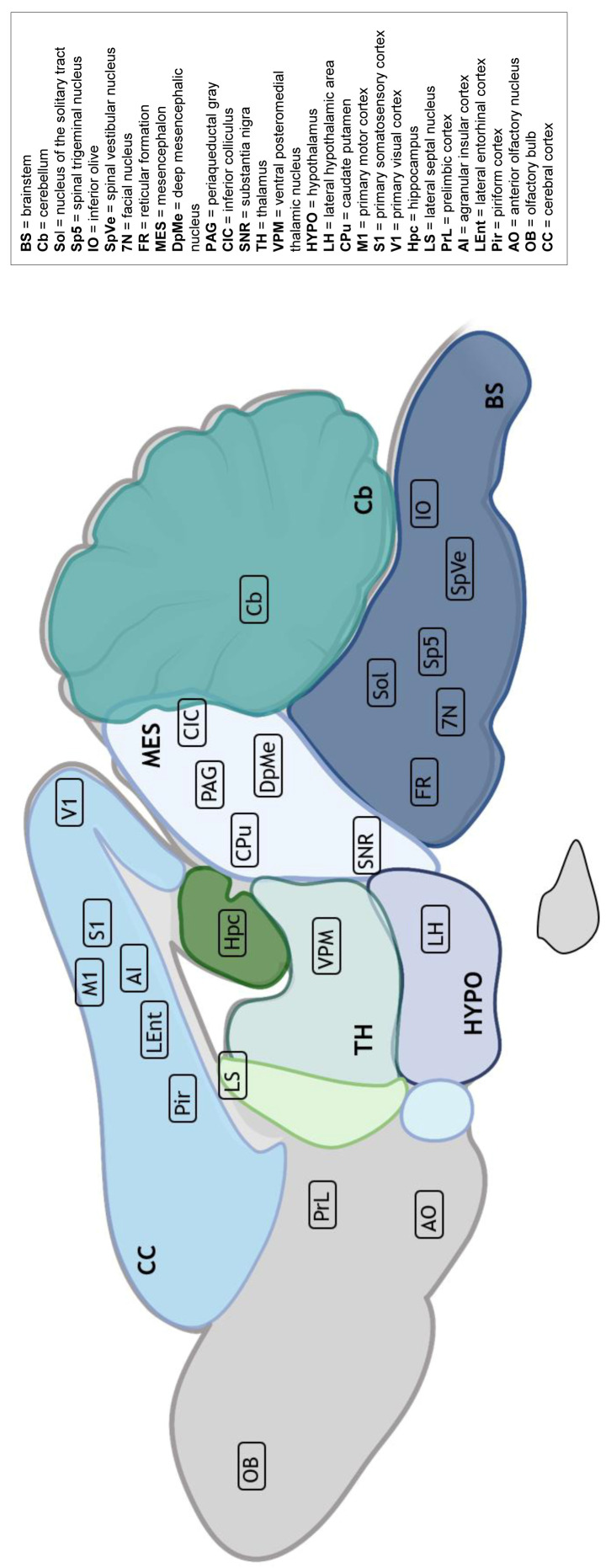
Cumulative anatomical distribution of LAT RNA detection in the murine brain following intranasal PrV-∆UL21/US3∆kin infection. LAT RNA was detected by RNAscope™ in multiple anatomical regions of the murine brain (brainstem, mesencephalon, diencephalon, telencephalon, and cerebellum) at 11–14, 28, 42, 105, and 190 dpi. The schematic sagittal brain section summarizes brain regions in which LAT-positive cells were identified in at least one animal at one or more time points during the observation period. The schematic representation illustrates the spatial extent of LAT detection over time and does not imply simultaneous, continuous or uniform expression across regions or time points. Created with BioRender.com.

LAT was detected on multiple CNS structures across time points (Figure 3). In the hindbrain, signals were observed in the Cb and brainstem (BS) nuclei including the nucleus of the solitary tract (Sol), inferior olive (IO), Sp5, spinal vestibular nucleus (SpVe), facial nucleus (7N), and the reticular formation (FR).

In the midbrain, LAT-positive signals were found in the substantia nigra (SNR), caudate putamen (CPu), deep mesencephalic nucleus (DpMe), and central nucleus of the inferior colliculus (CIC). Thalamic and hypothalamic areas such as the ventral posteromedial nucleus (VPM) and lateral hypothalamus (LH) showed expression.

In the telencephalon, signals were consistently detected in mesiotemporal regions including the Hpc, lateral septal nucleus (LS), and LEnt. Additional expression was observed in the Pir, AI, prelimbic cortex (PrL), and anterior olfactory nucleus (AO), as well as in primary visual (V1) and motor cortices (M1), S1 and in the OB.

### 3.4. Semi-Quantitative Analysis of UL19 and LAT Signals

**Figure 4:**
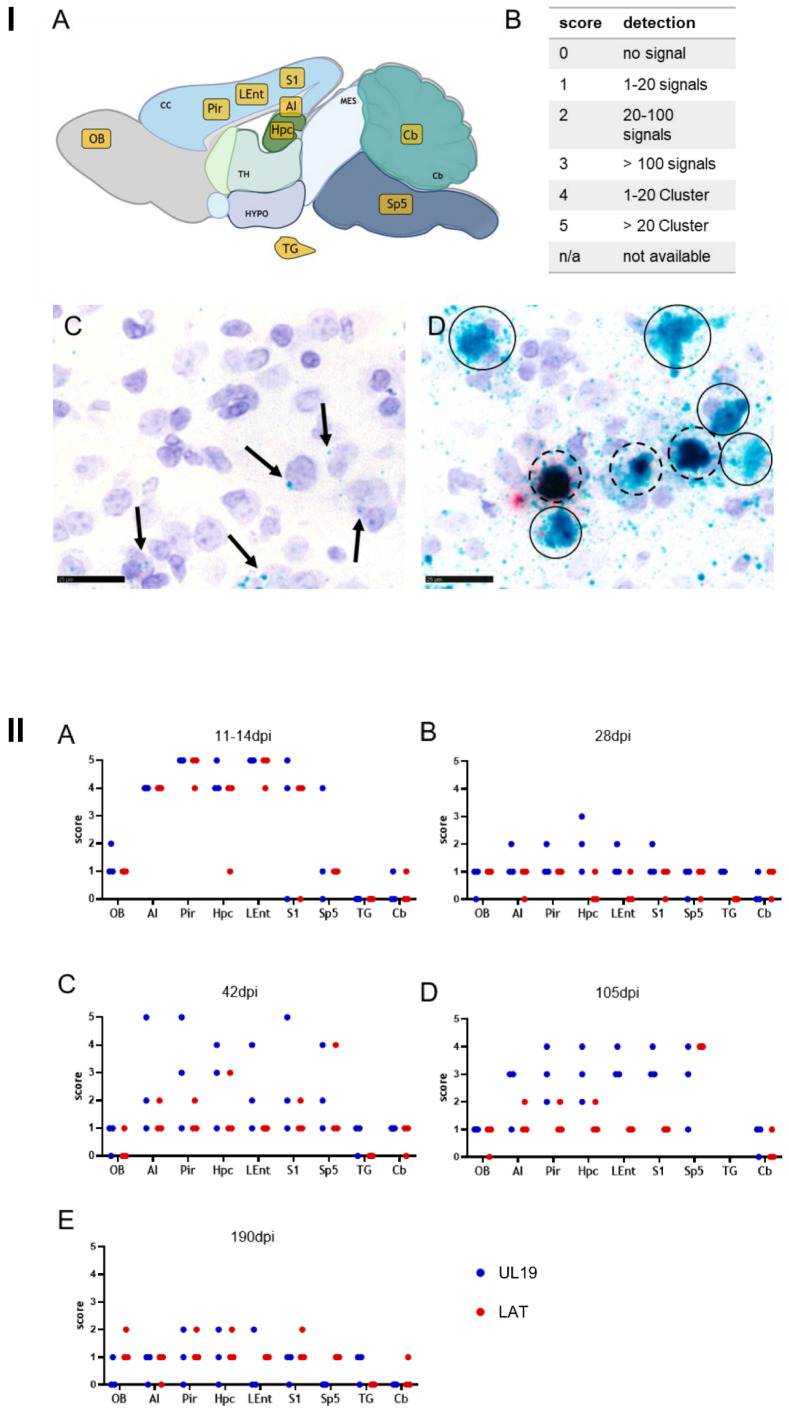
Detection of UL19 and LAT RNA transcripts in murine brain tissue using RNAScope™ in situ hybridization. (**I**) Methodological overview. (**IA**) Overview of the selected brain regions analyzed (yellow-shaded boxes). (**IB**) Scoring criteria for positive signal detection based on the number and clustering of signals within these regions (ROIs). Scoring was based on standardized coronal brain sections representing six anatomical levels (L1-6). Each ROI was evaluated at one or more anatomically defined levels (Figure S1). If multiple levels/ROI were available, the highest score per anatomical region was recorded. All evaluations were performed manually at high-power magnification (20× or 40×). (**IC**) Individual RNA transcripts appear as distinct chromogenic dots (arrow); green dots represent UL19 RNA transcripts. (**ID**) Accumulation of transcripts may result in clusters (solid circles), which can comprise different transcripts, indicated by mixed red (LAT) and green (UL19) signals (dashed circles). (**IC**,**ID**) show coronal sections of a murine brain at the level of the temporal lobe at 14 dpi with PrV-∆UL21/US3∆kin. Scale bar = 25 µm. (**II**) Semi-quantitative analysis of RNAScope™ signal detection in PrV-infected murine brain tissue. Positive signals for UL19 (blue) and LAT (red) RNA transcripts were scored at 11–14 dpi (**IIA**), 28 dpi (**IIB**), 42 dpi (**IIC**), 105 dpi (**IID**) and 190 dpi (**IIE**). Scores represent data from three mice per time point and brain region. TG = trigeminal ganglion, Cb = cerebellum, Sp5 = spinal trigeminal nucleus, S1 = primary somatosensory cortex, Hpc = hippocampus, LEnt = lateral entorhinal cortex, Pir = piriform cortex, AI = agranular insular cortex, OB = olfactory bulb. Created with BioRender.com.

To assess transcriptional dynamics, nine brain regions were selected for semi-quantitative analysis at defined post-infection timepoints. Selected regions included CNS regions targeted by HSV-1 and PrV, including Pir, AI, LEnt, Hpc, S1, Sp5 and OB [28,40]. The Cb served as a reference region due to its low susceptibility to alphaherpesviral infection [41]. The TG was included as a canonical latency site [1] (Figure 4(IA)).

**Figure 5:**
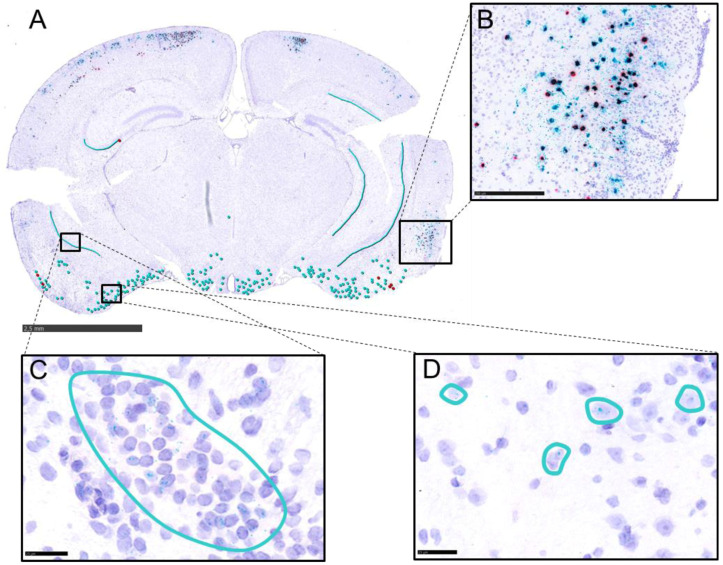
Detection of UL19 and LAT RNA transcripts during the acute phase (14 dpi) of PrV-∆UL21/US3∆kin infection using RNAscope™ in situ hybridization. (**A**) Coronal section of a PrV-∆UL21/US3∆kin-infected murine brain at the level of the temporal lobe showing UL19 (green dots) and LLT (red dots) RNA signals. Scale bar = 2.5 mm (**B**) Higher magnification of UL19 and LAT signals in LEnt. Scale bar = 250 µm. (**C**) Higher magnification of scattered UL19 signals in Hpc (cyan outline). Scale bar = 25 µm. (**D**) Higher magnification of UL19 signals in Pir (cyan circles). Scale bar = 25 µm. Hpc = hippocampus, LEnt = lateral entorhinal cortex, Pir = piriform cortex.

**Figure 6:**
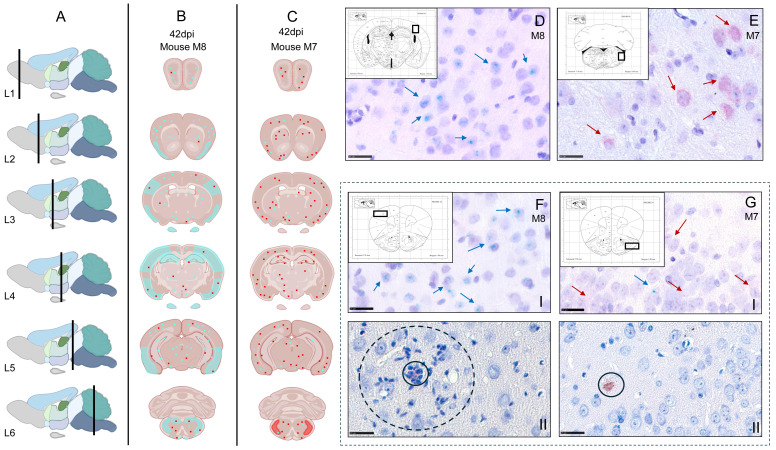
Regional distribution patterns of UL19 and LAT RNA signals across defined anatomical brain regions in selected mice at 42 dpi. (**A**) Sagittal view of the murine brain indicating the coronal section levels analyzed (L1-6). (**B**,**C**) Schematic representations of coronal brain sections illustrating the regional distribution of UL19 and LAT RNA signals in two representative animals at 42 dpi (mouse M8 in **B**; mouse M7 in **C**). Dots indicate single RNA transcripts, whereas shaded areas represent transcript clusters (red = LAT; cyan = UL19). (**D**) Higher magnification of the S1 in mouse M8 showing intense neuronal UL19 signals, with clusters indicated by arrows. (**E**) Higher magnification of the Sp5 in mouse M7, showing prominent neuronal LAT signals, with clusters indicated by arrows. (**F**) Higher magnification of the M1 in mouse M8, demonstrating strong neuronal UL19 signals (**I**). A distinct PrV gB-positive signal is present within the same region and is associated with inflammatory cells (**II**, dashed circle). (**G**) Higher magnification of the Pir in mouse M7 showing distinct neuronal LAT expression and occasional UL19-positive cells (**I**). A distinct PrV gB-positive signal is also detected within the same region (**II**). Sp5 = spinal trigeminal nucleus, S1 = primary somatosensory cortex, M1 = primary motor cortex, Pir = piriform cortex. Scale bar = 25 µm. Coronal brain sections shown with The Mouse Brain in Stereotaxic Coordinates by Paxinos and Franklin, 2001 [37]. Created with BioRender.com.

Signals were scored on a 0–5 scale (Figure 4(IB)), distinguishing discrete puncta (Figure 4(IC)) from clustered signal patterns (Figure 4(ID)). Results are summarized in Figure 4II and detailed per animal in Table S1. Representative RNAscope™ images are shown in Figure 5 and Figure 6.

Between 11 and 14 dpi, both UL19 and LAT transcripts were abundantly present (Figure 4(IIA)), particularly in temporal and frontoparietal regions (Figure 5A). Strong signals (Score 4–5) were observed in the LEnt (Figure 5B); Hpc (Figure 5C), Pir (Figure 5D), AI, S1. Sp5 and OB showed only a few signals (Score 1) (Figure 4(IIA)). In the Cb, only minimal or no signals (Score 0–1) were present, and no transcripts were detected in the TG (Figure 4(IIA)).

At 28 dpi, LAT signal detection was at the baseline across all regions (Score 0–1), with no signal in the TG. UL19 remained low in OB, Sp5, TG, and Cb, but was modestly elevated in the LEnt, Pir, AI, S1, and Hpc (Score 1–2) (Figure 4(IIB)).

At 42 dpi, LAT largely remained at the baseline, except in one animal showing increased signals (AI, Pir, S1: score 2; Hpc: score 3; Sp5: score 4). UL19 detection was very heterogenous. Cb, TG, and OB remained low (score 0–1), whereas AI, Pir, Hpc, LEnt, S1 and Sp5 varied widely (1–5) (Figure 4(IIC)). Representative animals illustrate this observation: Mouse M8 displayed UL19 clusters across regions (Figure 6B), while mouse M7 showed LAT clusters in Sp5 and increased signal frequency in AI, Pir, Hpc, and S1, with low UL19 expression, suggestive of latency (Figure 6C). Representative RNAscope™ images of clustered transcript signals in M8 (S1) and M7 (Sp5) are provided in Figure 6D and Figure 6E, respectively.

At 105 dpi, LAT remained at the baseline, except consistently elevated signals in Sp5 (Score 4). UL19 again showed heterogeneity, with OB and Cb low (score 0 and 1), and the highest variability in Sp5 (score 1–4), AI (1–3), Pir/Hpc (2–4), and LEnt/S1 (3–4) (Figure 4(IID)). No TG samples were available for this time point.

At 190 dpi (post-immunosuppression), LAT remained mostly at basal levels with isolated increases in S1, Hpc, Pir, and OB (score up to 2). UL19 was generally low (score 0–1), with isolated signals in Pir, Hpc, and Lent (up to score 2) (Figure 4(IIE)).

A shift in the intracellular distribution of viral transcripts was observed. During the acute phase, UL19 RNA and LAT were frequently detected within the same cell or in immediately adjacent cells with neuronal morphology. From 28 dpi onward and throughout the remainder of the observation period, UL19 RNA and LAT signals remained detectable in cells with neuronal morphology, but were confined to distinct cells without further co-localization.

### 3.5. Viral Antigen Detection at 42 dpi

Based on RNAscope™ transcript analyses, two representative mice at 42 days post-infection (M7 and M8) were further examined for expression of the viral glycoprotein gB by immunohistochemistry. In mouse M8, which exhibited strong UL19 transcript signals in M1 (Figure 6(FI)), PrV gB-positive staining was detected in individual cells within the same region. This antigen detection spatially coincided with areas characterized by high UL19 and low LAT transcript abundance. gB-positive cells in M1 were surrounded by inflammatory cells (Figure 6(FII)). In contrast, mouse M7 showed PrV gB immunoreactivity in a single cell within the Pir, a region showing abundant LAT and sporadic UL19 signals (Figure 6(GI)). In this case, gB-positive staining was not associated with an overt inflammatory infiltrate (Figure 6(GII)).

### 3.6. Detection of UL19 and LAT Transcripts by RT-qPCR

**Figure 7:**
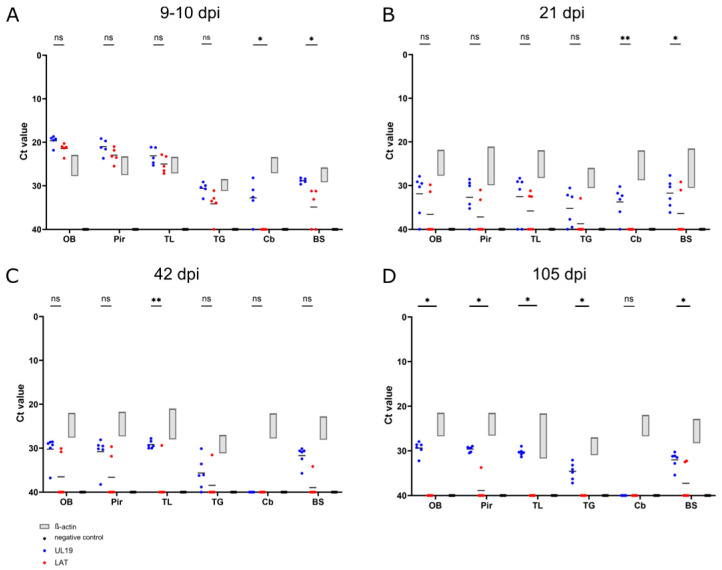
Detection of UL19 and LAT transcripts in selected brain regions using RT-qPCR. Transcript levels of the lytic gene UL19 and the LAT were assessed by RT-qPCR in brain tissue from six mice per time point, including mock-inoculated mice serving as negative controls. Tissue was collected at four time points: (**A**) 9–10 dpi (five animals euthanized at the humane endpoint), (**B**) 21 dpi, (**C**) 42 dpi, and (**D**) 105 dpi. Dissected brain regions included the olfactory bulb (OB), piriform cortex (Pir), temporal lobe (TL), trigeminal ganglion (TG), cerebellum (Cb) and brainstem (BS). Ct values are shown for each region, bars indicate the geometric mean per group. ß-actin was included as an internal control to verify RNA integrity and reverse transcription efficiency and to define a reliable detection range. * *p* < 0.05, ** *p* < 0.001, ns = non significant.

To complement RNAscope™ findings, RT-qPCR was performed to quantify transcripts of UL19 and LAT in six brain regions (OB, Pir, temporal lobe (TL), TG, Cb, BS) from mice sacrificed at 9–10 (humane endpoint), 21, 42 and 105 dpi. Ct values for UL19 and LAT are summarized in Figure 7, and corresponding clinical data are detailed in Figure S4.

Between 9 and 10 dpi, animals reaching the humane endpoint exhibited severe signs (hunching, seizures, >20% weight loss) (Figure S4). High transcript levels of both UL19 and LAT were detected in the OB, TL, and Pir, reflected by low Ct values (<25), which lay outside the β-actin-defined normalization range. In contrast, the TG, Cb, and BS showed higher Ct values (>30), indicating lower transcript abundance (Figure 7A).

At 21 dpi, both transcripts were detected only at low levels, with CT values close to the assay detection threshold (Ct > 35), and outside the normalized range. UL19 signals tended to be more frequently detectable than LAT, although this difference did not reach significance. Two mice (M25 and M24) showed comparatively lower LAT Ct values; but only one (M25) displayed clinical signs, including seizures and localized alopecia (Figure 7B and Figure S4).

At 42 dpi, UL19 transcripts were sporadically detectable in OB, TL, Pir, and BS (Ct > 30), but were absent Cb and TG. LAT expression was largely undetectable, except in one mouse (M29), which exhibited low-level expression across multiple regions except Cb (Figure 7C).

At 105 dpi, UL19 remained detectable at low levels in all regions (Ct 28–35) except Cb. LAT expression was largely absent; however, two animals (M36, M37) exhibited low-level expression in BS and Pir (Ct 32) (Figure 7D). Both animals showed only mild clinical signs, such as ruffled fur (Figure S4).

### 3.7. Histopathological Temporal Profiling

Histopathology was performed on consecutive sections of the same brain tissue samples analyzed by the RNAscope™.

Long-term histomorphological alterations in the CNS

**Figure 8:**
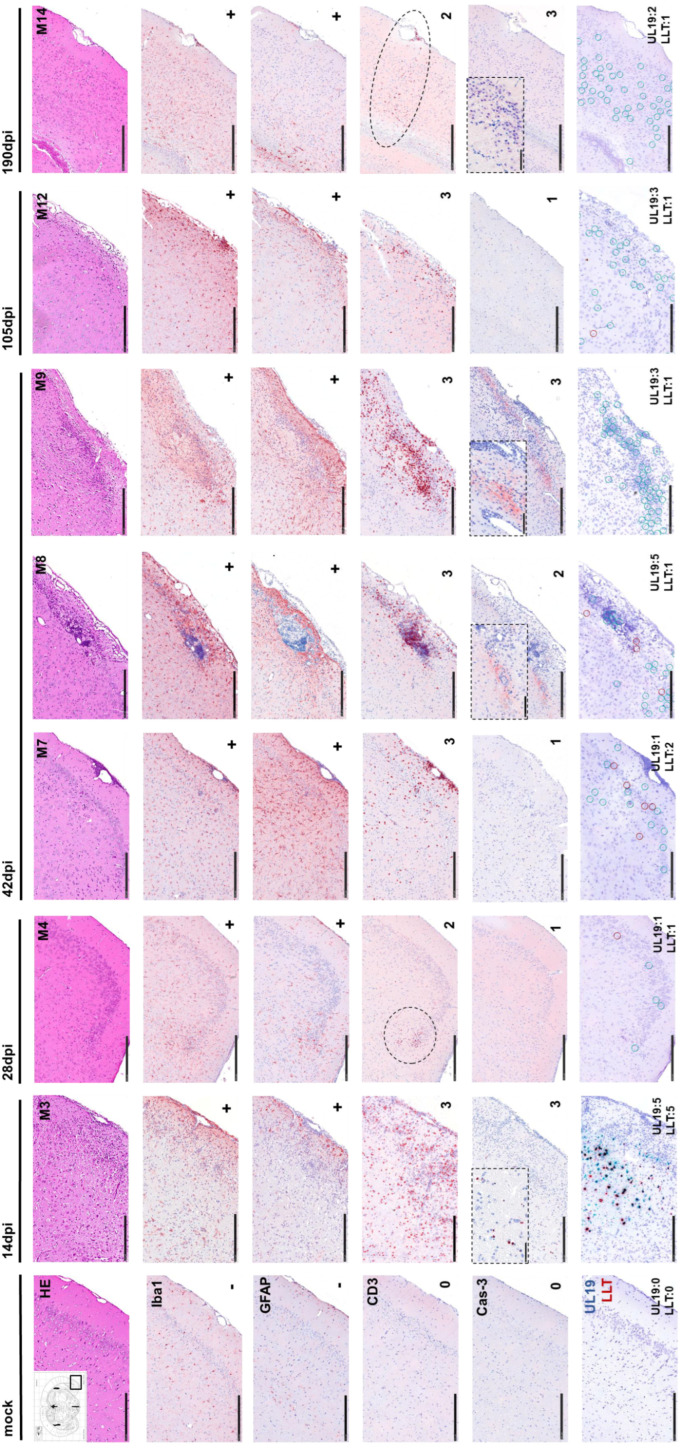
Representative images of long-term lesions in the temporal lobe (piriform cortex) from 14 to 190 dpi in PrV-∆UL21/US3∆kin-infected mice. Temporal progression of infection-associated neuropathology was assessed by histopathology (H&E), immunohistochemistry (Iba1, GFAP, CD3, cleaved caspase-3 (Cas-3), and RNAscope™ in situ hybridization targeting UL19 (lytic) and LAT (latency-associated) transcripts. Representative brain sections are shown for mice sacrificed at 14, 28, 105, and 190 dpi; three animals are shown for 42 dpi to illustrate inter-individual variability in histopathological and molecular findings. At 14 dpi, severe necrotizing meningoencephalitis was evident in the temporal lobe, accompanied by dense infiltrates of CD3^+^ T cells, Iba1^+^ microglia/infiltrating macrophages, reactive GFAP^+^ astrocytes, and abundant Cas-3^+^ apoptotic cells. From 28 to 105dpi, mild meningoencephalitis was detected, characterized primarily by CD3+ and Iba1+ immune cells. A gradual increase in GFAP expression indicated ongoing astrogliosis, while only a few Cas-3^+^ cells were detected, suggesting reduced apoptotic activity. At 42 dpi, mild-to-moderate meningoencephalitis was present, with pronounced CD3^+^ T-cell infiltration and inter-individual variability in Cas-3^+^ cell density ranging from low to high. At 190dpi, mild meningoencephalitis was still evident, characterized by ongoing CD3^+^, Iba1^+^ and GFAP^+^ immune cell infiltration together with a high number of Cas-3^+^ cells. Semi-quantitative scores from IHC and in situ hybridization analyses of the piriform cortex (ROI) are shown in the lower right corner of each panel. Scale bar = 250 µm.

Histomorphological changes were consistently observed throughout the entire observation period across the analyzed brain regions (Figure 8).

H&E staining revealed severe necrotizing meningoencephalitis during the acute phase, predominantly affecting mesiotemporal regions (piriform and prefrontal cortices). Lesions were associated with dense T-cell and macrophage infiltrates, neuronal necrosis, and marked gliosis. Between 28 and 190 dpi, pathology shifted to a milder phenotype. Low-grade meningoencephalitis persisted with scattered single-cell necrosis in mesiotemporal and frontal regions. Perivascular and meningeal T-cell/histiocyte infiltrates remained detectable, alongside ongoing glial activation (Table S1).

### 3.8. Spatiotemporal Patterns of T-Cell Infiltration and UL19 Transcripts

**Figure 9:**
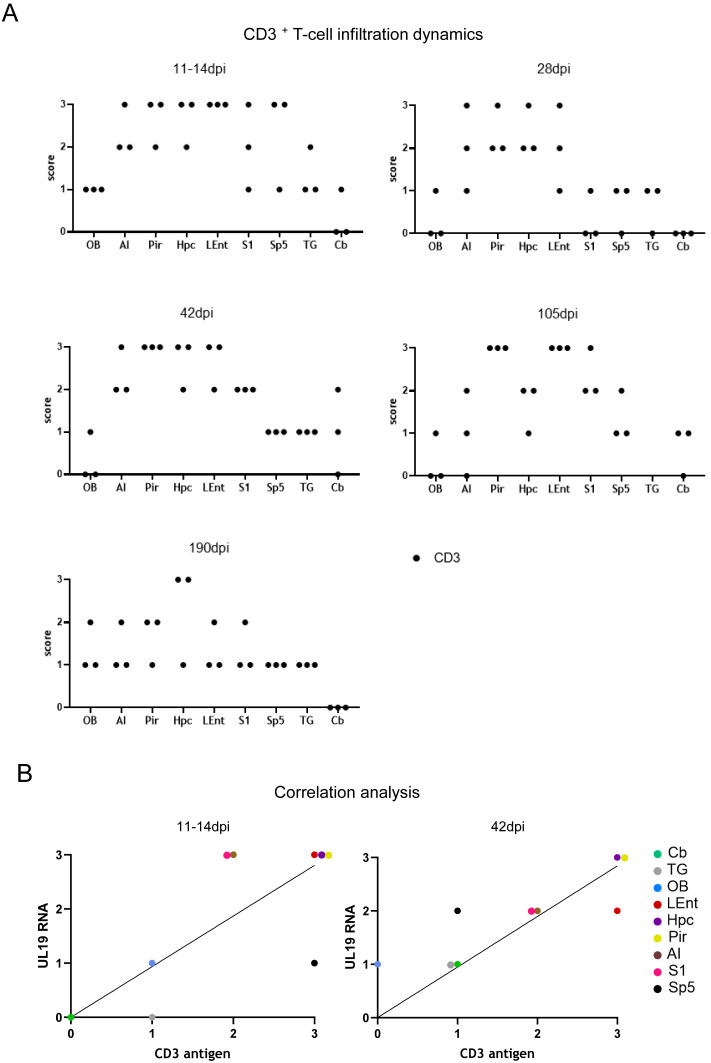
Semi-quantitative analysis of CD3^+^ T-cell infiltration and correlation with UL19 RNA in PrV-ΔUL21/US3Δkin-infected murine brain tissue. (**A**) Semi-quantitative scoring of CD3^+^ T-cell infiltration in selected brain regions at 11–14, 28, 42, 105 and 190 dpi. Scores from three mice per time point are shown. (**B**) Correlation analysis between CD3^+^ T-cell infiltration and UL19 RNA transcript detection. Mean CD3 antigen scores (x-axis) and mean UL19 RNA scores (y-axis) from three replicates per region were subjected to linear regression and Spearman’s correlation analysis. A significant positive correlation was observed at 11–14 dpi (*p* ≤ 0.05) and 42 dpi (*p* ≤ 0.01). TG = trigeminal ganglion, Cb = cerebellum, Sp5 = spinal trigeminal nucleus, S1 = primary somatosensory cortex, Hpc = hippocampus, LEnt = lateral entorhinal cortex, Pir = piriform cortex, AI = agranular insular cortex, OB = olfactory bulb.

Region-specific patterns of T-cell infiltration detected by CD3 staining were observed (Figure 9A). In Pir and LEnt, T-cell scores remained high (2–3) through 105 dpi, declining slightly (1–2) at 190 dpi. AI showed a similar trajectory with earlier decline. Hpc infiltration remained at score 2–3 at most time points, peaking at 190 dpi. S1 fluctuated, with high scores at 11–14, 42, and 105 dpi. Sp5 was largely unaffected, except for a transient peak at 11–14 dpi. TG, Cb, and OB consistently showed minimal infiltration. Correlation analysis demonstrated a strong positive association between CD3^+^ T-cell density and UL19 transcript detection, particularly at 11–14 dpi (*p* ≤ 0.05) and 42 dpi (*p* ≤ 0.01) (Figure 9B).

### 3.9. Integrated Spatiotemporal Profiling of Viral Transcription, Neuroinflammation, and Clinical Outcome

Representative images from the piriform cortex are shown in Figure 8.

During the acute phase (11–14 dpi), animals developed severe meningoencephalitis characterized by dense T-cell infiltrates (score 3), marked gliosis, and abundant apoptotic cells (Cas-3 score 3). Strong signals for both UL19 and LAT transcripts (score 5) were detected, coinciding with severe clinical manifestations.

At 28 dpi, inflammation had subsided to a mild meningoencephalitis, accompanied by ongoing gliosis. T-cell infiltration (score 2) and apoptotic activity (Cas-3 score 1) were lower, and viral transcript levels were low (score 1). Correspondingly, only minimal clinical signs were observed.

At 42 dpi, inter-individual variability became apparent. Mouse M7 showed mild meningoencephalitis characterized by dense T-cell infiltrates (score 3), marked gliosis and minimal apoptosis (Cas-3 score 1), low UL19 expression (score 1), and moderately increased LAT (score 2). In contrast, M8 exhibited moderate meningoencephalitis characterized by dense T-cell infiltrates (score 3), marked gliosis and moderate apoptosis (Cas-3 score 2), strong UL19 expression (score 5), and low LAT (score 1). Mouse M9 also showed moderate meningoencephalitis characterized by dense T-cell infiltrates (score 3), marked gliosis and pronounced apoptosis (Cas-3 score 3), moderate UL19 levels (score 3), low LAT (score 1), and behavioral abnormalities such as stargazing.

At 105 dpi, mild meningoencephalitis persisted, with high T-cell infiltration (score 3), moderate UL19 expression (score 3), and low LAT (score 1). Apoptosis remained minimal (Cas-3 score 1), while overt clinical signs were still present.

By 190 dpi, inflammation was mild, with ongoing T-cell infiltration (score 2) and low expression of UL19 (score 2) and LAT (score 1). Notably, apoptotic activity was elevated (Cas-3 score 3), despite the absence of clinical signs.

Mock-infected mice showed no abnormalities. An immunosuppressed mock control, however, exhibited increased apoptosis (Cas-3 score 3). A full summary of findings is provided in Table S1.

## 4. Discussion

In this study, we examined the long-term dynamics of PrV infection in the murine CNS following intranasal inoculation with the attenuated PrV-ΔUL21/US3Δkin. By integrating molecular, histopathological, and clinical analyses, we identified distinct spatiotemporal patterns of viral transcriptional activity associated with neuroinflammation and clinical outcomes. An additional immunosuppressed cohort enabled assessment of the potential for viral reactivation during prolonged infection.

Infected mice followed a multiphasic disease trajectory consistent with previous observations [29]. During the acute phase, animals developed severe clinical signs including seizures, hyperactivity, and “stargazing”, reflecting neuronal dysfunction particularly in mesiotemporal regions [42,43]. Mild-to-moderate clinical signs persisted in a subset of animals at later time points, and immunosuppression at 170 dpi triggered transient disease recurrence. These clinical features closely resemble the manifestations of human HSE, in which hippocampal and entorhinal involvement underlies cognitive deficits, memory impairment, and seizures [21,44,45].

RNAscope™ analysis demonstrated widespread neuronal permissiveness to long-term viral presence, with LAT und UL19 transcripts detected across the hindbrain, midbrain, diencephalon, and telencephalon, including mesiotemporal, olfactory, and neocortical regions. Semi-quantitative assessment revealed distinct temporal dynamics: during the acute phase (11–14 dpi), both LAT and UL19 transcripts were abundant in mesiotemporal and frontoparietal areas; by 28 dpi, both signals declined markedly; and from 42 dpi onward, LAT persisted at low baseline levels, whereas UL19 displayed pronounced inter-individual heterogeneity. Following immunosuppression at 190 dpi, LAT levels remained low, while UL19 expression increased modestly in selected regions.

RT-qPCR analyses supported the overall dynamics observed by RNAscope™, showing the highest viral transcript levels in the olfactory bulb, temporal lobe, and piriform cortex during the acute phase, followed by markedly lower but still detectable UL19 signals at later time points. Discrepancies between RNAscope™ and RT-qPCR results, particularly in the OB, likely reflect methodological differences, as RT-qPCR was performed on homogenized tissue samples, whereas RNAScope™ provides single-cell and single-section resolutions. While UL19 transcripts were detectable by RT-qPCR across multiple regions and time points, reliable detection of LAT by RT-qPCR was not achieved under the conditions applied, most likely due to its low abundance during long-term latency. Accordingly, the RNAscope™ proved more sensitive for detecting sparse, cell-restricted viral transcripts, whereas RT-qPCR provides complementary information on overall viral transcriptional activity at the tissue level.

Unexpectedly, LAT signals were largely absent from the TG, although this structure is permissive for the PrV mutant [28] and is considered the primary alphaherpesvirus latency reservoir [1,10,46]. Instead, brainstem regions, especially the Sp5, consistently exhibited LAT expression at later time points. This observation aligns with reports suggesting that reactivation may occur more readily in brainstem neurons than in the TG [19,20].

The preferential involvement of specific brain regions raises important mechanistic questions regarding viral entry, neuronal susceptibility, and immune control. One contributing factor may be regional differences in expression of alphaherpesvirus entry receptors such as nectin-1 [2,47], which, although broadly expressed in the CNS, shows regional variability [48,49]. In line with this, prior work using the PrV-ΔUL21/US3Δkin model demonstrated that cortical vulnerability is shaped by the neuronal subtype and nectin-1 distribution, with regions with high receptor expression correlating with viral antigen detection [50]. Cell-intrinsic susceptibility further contributes to this pattern, as primary cerebral neurons display higher permissiveness than cerebellar neurons in vitro [50]. Similar region-specific tropism has been described for other neurotropic viruses, such as West Nile virus [51], and may be linked to baseline differences in interferon-stimulated genes [52].

At the single-cell level, LAT and UL19 were frequently co-detected during the acute phase, indicating transient overlap of lytic and latency-associated transcription. From 28 dpi onward, however, transcripts were strictly segregated to distinct neurons, with no further co-localization. This shift suggests an early overshooting response that subsequently resolves into mutually exclusive transcriptional programs. A similar phenomenon has also been described previously [53], and is consistent with evidence that lytic and latency-associated gene expression can transiently coexist in the same cell [54,55,56,57].

UL19 expression showed pronounced inter-individual variability at 42 and 105 dpi. Some animals displayed robust UL19 signals with low-level LAT expression across multiple regions, consistent with episodic viral reactivation, paralleling findings by Menendez et al. [58], who detected lytic HSV-1 activity beyond 60 dpi. Other animals predominantly displayed LAT signals with minimal UL19 RNA detection. This transcriptional pattern is consistent with a latency-like state; however, LAT transcripts are also produced at low levels during lytic infection [55,57]. Such heterogeneity likely reflects differences in latent genome copy numbers, which have been shown to correlate with reactivation frequency [59,60]. At 42 dpi, viral glycoprotein gB was detected in single cells within regions of high UL19 RNA detection, and was accompanied by infiltrating immune cells, supporting active or recent reactivation events. Notably, gB immunoreactivity was also observed in a single cell from an animal otherwise displaying a transcriptional pattern dominated by LAT, without detectable immune cell infiltration. This finding is compatible with a very recent or spatially restricted reactivation event that may precede immune cell recruitment.

The rare and spatially restricted detection of viral proteins further raises the possibility of abortive reactivation events—defined as the initiation of lytic transcription without progression to productive viral replication. In such scenarios, lytic transcripts may be detectable while viral protein expression remains limited, suggesting premature interruption of the lytic program. This restricted protein expression likely reflects potent intrinsic antiviral mechanisms within neurons, which can sense and actively suppress viral gene expression [61].

Histopathological analysis confirmed persistent CNS inflammation. During the acute phase, severe necrotizing meningoencephalitis affected mesiotemporal, piriform, and prefrontal regions. At later stages, mild but sustained lymphohistiocytic meningoencephalitis, gliosis, and focal neuronal necrosis was present up to end of the experiment (190 dpi). T-cell infiltration followed region-specific patterns and correlated with UL19 RNA detection, particularly at 11–14 and 42 dpi, supporting the concept that T-cell clusters mark sites of localized viral transcriptional activity and potential reactivation sites [46]. CD8^+^ T cells are known to suppress reactivation in an antigen-specific manner [56], and LAT transcripts may counteract this by inhibiting apoptosis through caspase-3 regulation [62]. Consistent with this, cleaved caspase-3 was frequently detected in regions with high UL19 RNA signals and pronounced T-cell infiltration.

The immunosuppressed cohort provided additional insights into potential reactivation dynamics. Although cyclophosphamide/dexamethasone treatment was followed by moderate clinical signs, only low-level UL19 RNA signals were detected at the time of analysis, and increased caspase-3 activity was observed in both infected and control animals. Thus, although the transient clinical deterioration points to reactivation, direct molecular or histopathological evidence of active viral reactivation could not be demonstrated. It is therefore possible that reactivation occurred transiently and was no longer detectable at the time of euthanasia, potentially due to the timing of sampling. Future studies with shorter treatment-to-sampling intervals will be required to capture immediate reactivation events more precisely.

Finally, the use of an attenuated viral PrV mutant must be considered when extrapolating these findings. Although PrV-ΔUL21/US3Δkin differs from the wild-type strains in replication kinetics, it retains key neuroinvasive properties and targets similar brain regions [28]. Importantly, infection with wild-type PrV leads to rapid death of the infected mice, precluding an analysis of the immune response and long-term viral dynamics [28,63]. Thus, the attenuated model uniquely enables the investigation of prolonged viral transcriptional activity and CNS immune surveillance.

## 5. Conclusions

Our study demonstrates that our long-term pseudorabies virus infection model, with its striking similarity to HSE in humans [28], is characterized by region-specific and temporally dynamic viral transcriptional activity, accompanied by persistent neuroinflammation in the murine CNS. Viral transcription was not restricted to classical peripheral sites of latency such as the TG, but was detected across multiple CNS regions, particularly within mesiotemporal structures. The close spatial association between lytic transcript detection and T-cell infiltration suggests ongoing immune surveillance of localized viral activity and is compatible with abortive and/or episodic reactivation events rather than continuous productive infection. By integrating molecular, histopathological, and clinical readouts, this model provides a robust framework for studying long-term viral transcriptional activity and immune regulation within the CNS during neurotropic herpesvirus infection.

## Figures and Tables

**Table 1:** Primary antibodies used for immunohistochemistry.

Antigen	Target	Manufacturer	Clonality/Host Species	Working Dilution
Iba 1	microglia/macrophages	FUJIFILM Wako, 01-19741	polyclonal rabbit	1:500
GFAP	astrocytes	Abcam, ab16997	polyclonal rabbit	1:400
CD3	t-cells	DAKO, A0452	polyclonal rabbit	1:100
cleaved caspase-3 (Cas-3)	apoptosis	Cell Signaling Technology, 9661	polyclonal rabbit	1:800
PrVgB	viral glycoprotein gB	FLI Riems	polyclonal rabbit	1:2000

**Table 2:** Semi-quantitative scoring of CD3 and Cas-3 immunoreactivity in murine brain sections. The scoring system was applied to assess CD3^+^ T-cell infiltration and Cas-3^+^ apoptotic cells across brain regions. Immunostaining for CD3 and Cas-3 was performed on coronal brain sections representing six anatomical levels. Selected ROIs were evaluated; if multiple levels/ROI were available, the highest score per region was used. Scores ranged from 0 to 3 and were assigned according to the number of positive cells detected by light microscopy.

Score	CD3/Cas-3
0	absent
1	<10 cells
2	11–20 cells
3	>20 cells

**Table 3:** Qualitative assessment of Iba1 and GFAP immunoreactivity in murine brain sections. Immunostaining for Iba1 and GFAP was performed on coronal brain sections representing six anatomical levels. ROIs were analyzed at these levels; if multiple levels/ROI were available, the highest score per region was recorded. Glial activation was assessed qualitatively and categorized as present (+) or absent (−), based on the presence of immunoreactive cells with reactive morphology.

Score	Iba-1/GFAP
−	absence of glial activation
+	glial activation/cluster

**Table 4:** Primer sequences and characteristics for RT-qPCR detection of LAT and UL19 transcripts. Primer pairs were designed for RT-qPCR analysis targeting the LAT and the lytic viral gene UL19 of PrV-ΔUL21/US3Δkin. The table lists primer sequences, expected amplicon sizes, GC contents and melting temperatures (Tm) used for specific amplification.

Name	Sequence (5′-> 3′)	Location *	Size [bp]	G+C Content [%]	Annealing Temperature
Lat2 Probe (411)	[FAM] GTC TTC ACC CCA GAT GAC CG [TAM]	95.994–96.013	20	60	61.4 °C
LAT2 323 F	AGT TGA AGA CGG GGA CTC TG	95.906–95.925	20	55	59.4 °C
LAT2 470 R	GTC GAC GGG GAA GAG GAT GA	96.034–96.053	20	60	61.4 °C
LAT2-template (Oligomer)	CAG TTG AAG ACG GGG ACT CTG GGG CGG GCG CGA GAC CCA GAC CCG GAG CCC TGC CCT TCG GCC TCC TCG TGG CGC ACC TCC TCG GTA TAG TCT TCA CCC CAG ATG ACC GCG AAG CCC CCC CCT ACC GGC TCA TCC TCT TCC CCG TCG ACA	-	150	68	-
PrV-UL19-TEX	CGC AAC ACG CAC AAC GCC GCC	67.510–67.530	21	71.4	67.6
PrV-UL19 1817F	CGC AGT GCA TCC AGA GCT AC	67.487–67.506	20	60	61.4
PrV-UL19 1966R	CGT TGC CCA GGT AGG TGT TG	67.563–67.582	20	60	61.4

* GenBank accession number: JF797218 (Suid herpesvirus 1 strain Kaplan, complete genome).

## Data Availability

All data generated or analyzed during this study are included in this published article and its Supplementary Information Files.

## Supplementary Materials

The following supporting information can be downloaded at https://www.mdpi.com/article/10.3390/pathogens15040395/s1, Figure S1: Delineation of brain regions for semi-quantitative analysis of immunohistochemistry and in situ hybridization. Anatomical boundaries of each brain region were defined according to The Mouse Brain in Stereotaxic Coordinates (Paxinos & Franklin, 2001) [37]. Colored overlays indicate the specific areas analyzed within coronal sections at levels 1–6. Yellow: olfactory bulb (OB); light green: primary somatosensory cortex (S1); orange: agranular insular cortex (AI); blue: piriform cortex (Pir); grey: trigeminal ganglion (TG); pink: hippocampus (Hpc); purple: lateral entorhinal cortex (LEnt); brown: cerebellum (Cb); dark green: spinal trigeminal nucleus (Sp5). Brain atlas images adapted from The Mouse Brain in Stereotaxic Coordinates (Paxinos & Franklin, 2001) [37]. Figure S2: Representative images of RNAscope™ detection in the murine brain. (A) Negative control probe targeting Escherichia coli DapB shows absence of specific signals, confirming assay specificity. (B) Positive control probes for murine Ppib (C1, green) and Ubc (C2, red) demonstrate robust and widespread expression in neuronal tissue, validating RNA integrity and hybridization efficiency. Scale bar = 250 µm. Figure S3: Experimental timeline of necropsies and tissue collection. Six- to eight-week-old CD1 mice were intranasally inoculated with PrV-∆UL21/US3∆kin (day 0) in two independent studies. In study 1, tissue samples were collected at 21, 42, and 105 dpi (indicated by stars). In study 2, mice received an immunosuppressive treatment (cyclophosphamide/dexamethasone) at 170 dpi, and tissue was collected at 28 dpi and 190 dpi. During the acute infection phase (11–14 dpi), mice that reached the humane endpoint were euthanized, and tissues were collected for analysis. Created with BioRender.com. Figure S4: Clinical condition of PrV-infected mice at euthanasia and corresponding heatmaps of UL19 and LAT gene expression across defined brain regions. (A) The clinical condition of each mouse at the time of euthanasia is shown in relation to corresponding gene expression. Mice marked with an asterisk (*) exhibited severe clinical signs and reached predefined humane endpoint criteria, necessitating euthanasia. (B,C) RT-qPCR was performed to quantify RNA transcripts of UL19 (B) and LAT (C) of PrV in selected brain regions (OB = olfactory bulb, Pir = piriform cortex, TL = temporal lobe, TG = trigeminal ganglion, Cb = cerebellum, BS = brainstem) at defined time points. Heatmaps were generated from Ct values to illustrate spatial and temporal patterns of viral gene expression. Lower Ct values (darker color intensity) indicate higher transcript abundance. Table S1: Summary of clinical findings, histopathological diagnoses, and molecular analyses. Each mouse is listed with clinical signs, HE-based pathological evaluation, and corresponding scores from IHC and RNAscope™ (ISH). Animals that reached the humane endpoint are indicated with an asterisk (*). TG = trigeminal ganglion, Cb = cerebellum, Sp5 = spinal trigeminal nucleus, S1 = primary somatosensory cortex, Hpc = hippocampus, LEnt = lateral entorhinal cortex, Pir = piriform cortex, AI = agranular insular cortex, OB = olfactory bulb.

## Abbreviations

The following abbreviations are used in this manuscript:

7N	facial nucleus
AD	Alzheimer’s disease
AI	agranular insular cortex
aMCI	amnestic mild cognitive impairment
Amp	amplicon
AO	anterior olfactory nucleus
AP	alkaline phosphatase
BS	brainstem
Cas-3	cleaved caspase-3
Cb	cerebellum
CIC	central nucleus of the inferior colliculus
CNS	central nervous system
CPu	caudate putamen
Ct	threshold cycle
dpi	days post-infection
DpMe	deep mesencephalic nucleus
dpt	days post-treatment
FCS	fetal calf serum
FFPE	formalin-fixed paraffin-embedded
Fig.	figure
FR	reticular formation
H&E	hematoxylin and eosin
Hpc	hippocampus
HRP	horseradish peroxidase
HSE	herpes simplex encephalitis
HSV-1	Herpes Simplex Virus Type 1
IO	inferior olive
LAT	latency-associated transcript
LEnt	lateral entorhinal cortex
LH	lateral hypothalamus
LLT	large-latency transcript
LS	lateral septal nucleus
M1	primary motor cortex
MEM	minimum essential medium
OB	olfactory bulb
PBS	phosphate-buffered saline
PFA	paraformaldehyde
Pir	piriform cortex
PrL	prelimbic cortex
PrV	Pseudorabies Virus
RK13	rabbit kidney cells
ROI	region of interest
RT	room temperature
RT-qPCR	quantitative real-time PCR
S1	primary somatosensory cortex
SNR	substantia nigra
Sol	nucleus of the solitary tract
Sp5	spinal trigeminal nucleus
SpVe	spinal vestibular nucleus
TBS	Tris-buffered saline
TG	trigeminal ganglion
TL	temporal lobe
Tm	melting temperatures
V1	primary visual cortex
VPM	ventral posteromedial nucleus
