# Supplementary material for: Regional and Temporal Patterns of Long-Term Pseudorabies Virus Detection and Neuropathology in the Murine CNS
Source: Pathogens. 2026 Apr 7;15(4):395. doi: 10.3390/pathogens15040395 (PMC13118813; doi:10.3390/pathogens15040395)
Supplement: Supplementary file 1 [file pathogens-15-00395-s001.zip › Figure S1.pdf]

Paxinos G., Franklin K., The Mouse Brain in Stereotaxic Coordinates, second edition, Academic Press, 2001

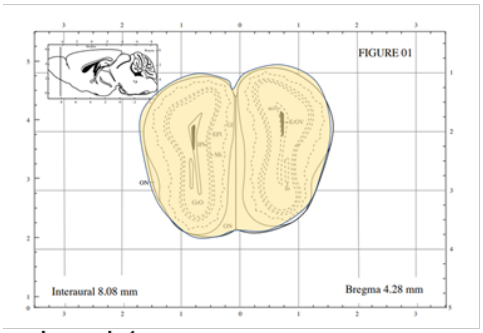

Level 1

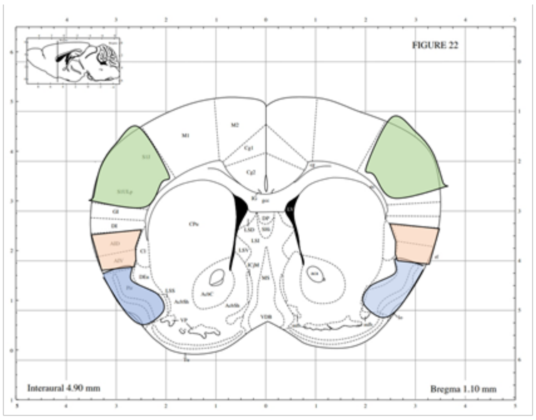

Level 2

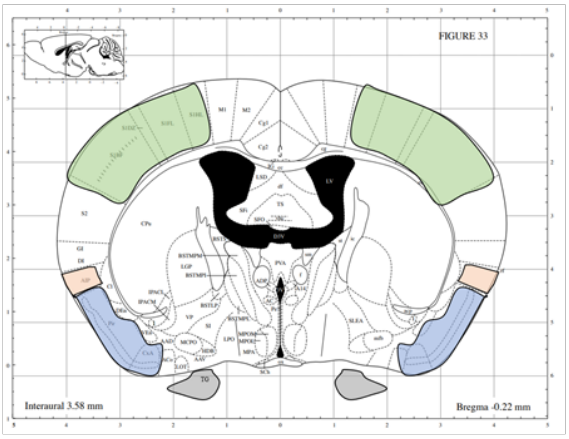

Level 3

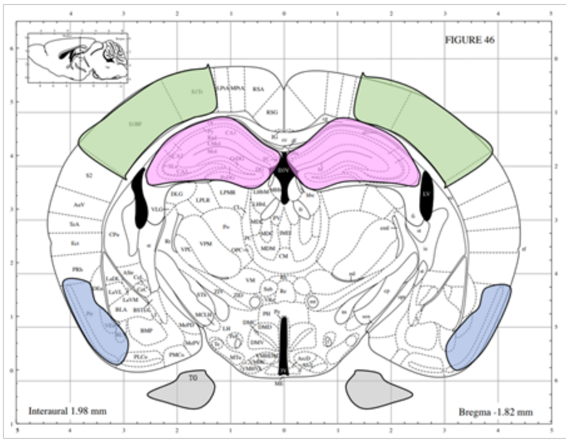

Level 4

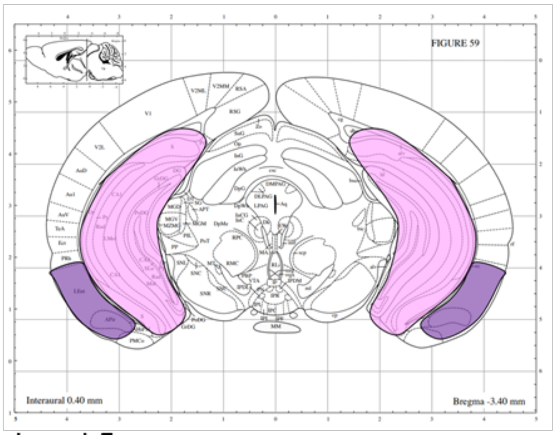

Level 5

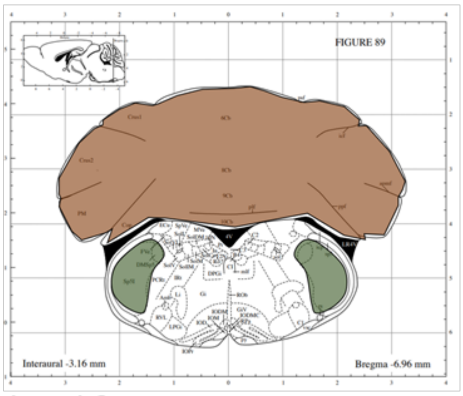

Level 6
