# Supplementary material for: Regional and Temporal Patterns of Long-Term Pseudorabies Virus Detection and Neuropathology in the Murine CNS
Source: Pathogens. 2026 Apr 7;15(4):395. doi: 10.3390/pathogens15040395 (PMC13118813; doi:10.3390/pathogens15040395)
Supplement: Supplementary file 1 [file pathogens-15-00395-s001.zip › Figure S4.pdf]

A

mouse	brain region	Ct value [LAT]	Ct value [UL19]	euthanasia [dpi]	clinical signs at time point of euthanasia
**M18***	OB	20,95	19,22	10	Severely hunched back, weight loss >20%, moderate pruritus, seizure with recovery
	Pir	23,48	21,55		
	TL	25,47	23,59		
	TG	40	32,97		
	Cb	40	33,36		
	BS	40	29,63		
**M19***	OB	21,31	19,05	10	seizure with recovery, severe hair loss (cheek)
	Pir	21,82	19,68		
	TL	23,24	21,2		
	TG	33,51	30,69		
	Cb	40	28,15		
	BS	31,18	28,79		
**M20***	OB	21,09	19,51	10	severely hunched back, weight loss >20%
	Pir	20,98	19,12		
	TL	22,82	21,11		
	TG	31,09	29,11		
	Cb	40	32,57		
	BS	40	29,05		
**M21***	OB	23,65	21,81	10	seizure with recovery, severely hunched back
	Pir	25,46	23,67		
	TL	27,14	25,27		
	TG	32,87	30,34		
	Cb	40	40		
	BS	33,09	28,38		
**M22***	OB	20,26	18,65	9	severely hunched back
	Pir	23,21	21,18		
	TL	26,49	24,66		
	TG	33,9	29,94		
	Cb	40	30,92		
	BS	31,21	28,16		
**M23**	OB	40	40	21	-
	Pir	40	40		
	TL	40	40		
	TG	40	39,5		
	Cb	40	35,98		
	BS	40	36,15		
**M24**	OB	40	36,25	21	'stargazing', mild hunched back, ruffled fur
	Pir	40	35,24		
	TL	31,18	28,31		
	TG	40	37,71		
	Cb	40	40		
	BS	40	34,5		
**M25**	OB	31,39	29,5	21	focal seizures, focal hair loss on the head
	Pir	30,98	29,39		
	TL	32,43	29,17		
	TG	32,91	30,53		
	Cb	40	31,73		
	BS	31,02	29,56		
**M26**	OB	40	30,27	21	Unilateral blepharitis
	Pir	40	30,04		
	TL	40	30,83		
	TG	40	40		
	Cb	40	32,31		
	BS	40	32,96		
**M27**	OB	40	29,12	21	-
	Pir	40	34,24		
	TL	40	40		
	TG	40	32,56		
	Cb	40	33,13		
	BS	40	30,27		
**M28**	OB	29,82	27,86	21	-
	Pir	33,23	28,54		
	TL	32,51	29,09		
	TG	40	32,17		
	Cb	40	30,2		
	BS	29,15	27,66		

**M29**	OB	30,81	28,59	42	-
	Pir	29,62	28,07		
	TL	29,36	27,76		
	TG	31,52	30,09		
	Cb	40	40		
	BS	34,15	30,59		
**M30**	OB	40	29,24	42	-
	Pir	40	29,49		
	TL	40	29,37		
	TG	40	33,58		
	Cb	40	40		
	BS	40	30,1		
**M31**	OB	40	29,93	42	-
	Pir	40	29,34		
	TL	40	29,99		
	TG	40	40		
	CB	40	40		
	BS	40	30,61		
**M32**	OB	40	28,53	42	Focal hair loss on the nasal bridge and flank
	Pir	40	30,13		
	TL	40	28,41		
	TG	40	36,08		
	Cb	40	40		
	BS	40	32,42		
**M33**	OB	40	36,75	42	-
	Pir	40	38,25		
	TL	40	29,99		
	TG	40	38,84		
	Cb	40	40		
	BS	40	35,7		
**M34**	OB	30,06	28,91	42	-
	Pir	31,83	30,29		
	TL	32.37	29,51		
	TG	40	36,21		
	Cb	40	40		
	BS	40	30,86		
**M35**	OB	40	28,59	105	nasal bridge edema
	Pir	40	29,17		
	TL	40	31,33		
	TG	40	33,19		
	Cb	40	40		
	BS	40	31,1		
**M36**	OB	40	29,72	105	ruffled fur
	Pir	33,72	29,41		
	TL	40	30,37		
	TG	40	32,05		
	Cb	40	40		
	BS	32,26	30,23		
**M37**	OB	40	29,24	105	-
	Pir	40	30,46		
	TL	40	30,28		
	TG	40	36,28		
	Cb	40	40		
	BS	32,46	32,79		
**M38**	OB	40	27,91	105	-
	Pir	40	29,37		
	TL	40	28,95		
	TG	40	34,13		
	Cb	40	40		
	BS	40	31,2		
**M39**	OB	40	28,67	105	-
	Pir	40	30,22		
	TL	40	30,73		
	TG	40	34,81		
	Cb	40	40		
	BS	40	31,47		
**M40**	OB	40	32,22	105	-
	Pir	40	28,95		
	TL	40	30,09		
	TG	40	37,19		
	Cb	40	40		
	BS	40	35,42		

B

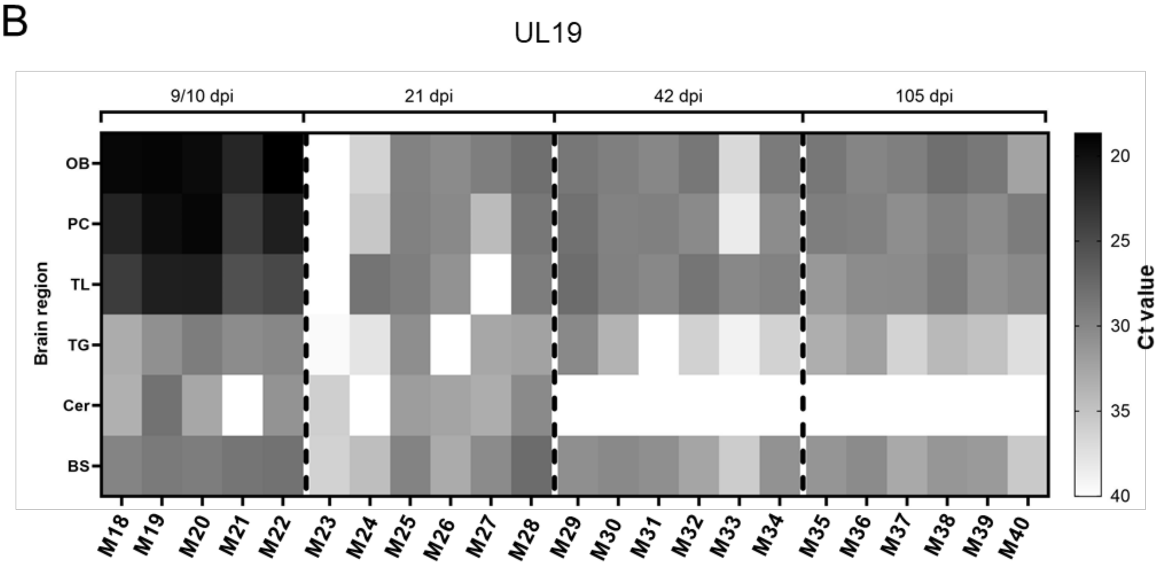
UL19
9/10 dpi
21 dpi
42 dpi
105 dpi
OB
PC
TL
TG
Cer
BS
Brain region
M18
M19
M20
M21
M22
M23
M24
M25
M26
M27
M28
M29
M30
M31
M32
M33
M34
M35
M36
M37
M38
M39
M40
Ct value
20
25
30
35
40

C

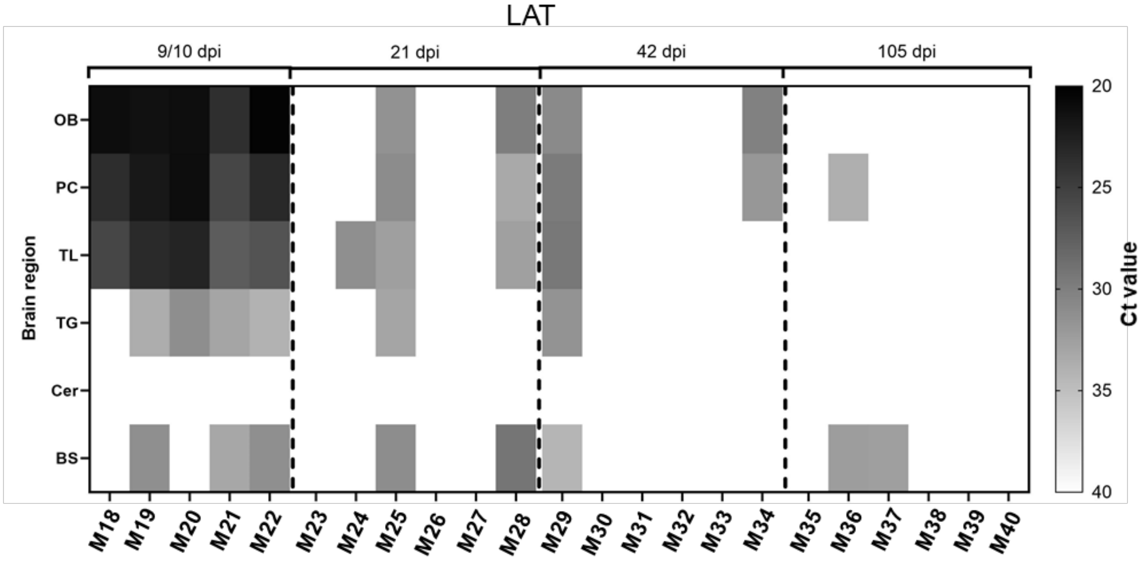
LAT
9/10 dpi
21 dpi
42 dpi
105 dpi
OB
PC
TL
TG
Cer
BS
Brain region
M18
M19
M20
M21
M22
M23
M24
M25
M26
M27
M28
M29
M30
M31
M32
M33
M34
M35
M36
M37
M38
M39
M40
Ct value
20
25
30
35
40
