# Supplementary material for: Regional and Temporal Patterns of Long-Term Pseudorabies Virus Detection and Neuropathology in the Murine CNS
Source: Pathogens. 2026 Apr 7;15(4):395. doi: 10.3390/pathogens15040395 (PMC13118813; doi:10.3390/pathogens15040395)
Supplement: Supplementary file 1 [file pathogens-15-00395-s001.zip › Table S1.pdf]

mouse	inoculum, treatment	euthanasia [dpi]	clinical signs at euthanasia day	H&E	IHC/ RNAScope™ (ISH) Scoring/ brain region		OB	AI	Pir	Hpc	LEnt	S1	Sp5	TG	Cb
M1	PrV-ΔUL21/US3Δkin	11*	severely hunched back, ruffled and dull fur, hemorrhagic skin erosions, apathy	severe meningoencephalitis with extensive neuronal necrosis in Pir, Hpc and LEnt, meningeal and perivascular infiltrates of T-lymphocytes and histiocytes as well as glial activation in the temporal lobe (Pir, Hpc, LEnt), prefrontal cortex (AI) and Sp5	IHC	CD3	1	2	2	3	3	1	3	2	0
						Iba1	+	-	-	+	+	-	+	-	-
						GFAP	+	-	-	-	+	-	+	-	-
					ISH	LLT	1	4	5	4	5	4	1	0	0
						UL19	2	4	5	5	5	4	1	0	0
M2	PrV-ΔUL21/US3Δkin	14*	severely hunched back, ruffled and soiled fur, hemorrhagic skin erosions, seizure with recovery, moderate pruritus	severe meningoencephalitis with extensive neuronal necrosis in Pir and LEnt, meningeal and perivascular infiltrates of T-lymphocytes and histiocytes as well as glial activation in the temporal lobe (Pir, Hpc, LEnt), parietal lobe (S1), prefrontal cortex (AI) and Sp5	IHC	CD3	1	2	3	2	3	2	3	1	0
						Iba1	-	-	+	+	+	-	+	-	-
						GFAP	-	-	-	+	+	-	+	-	-
					ISH	LLT	1	4	4	4	4	0	1	0	0
						UL19	1	4	5	4	5	0	0	0	0
M3	PrV-ΔUL21/US3Δkin	14*	moderately hunched back, ruffled and dull fur, neck hair loss, nasal bridge edema, seizure with recovery	severe meningoencephalitis with extensive neuronal necrosis in S1, AI, Pir, Lent and Hpc, meningeal and perivascular infiltrates of T-lymphocytes and histiocytes as well as glial activation in the temporal lobe (Pir, Hpc, LEnt), parietal lobe (S1) and prefrontal cortex (AI)	IHC	CD3	1	3	3	3	3	3	1	1	1
						Iba1	-	+	+	+	+	+	+	-	-
						GFAP	-	+	+	+	+	+	-	-	-
						Cas3	0	3	3	2	3	1	0	0	0
					ISH	LLT	1	4	5	1	5	4	1	0	1
						UL19	1	4	5	4	5	5	4	0	1
M4	PrV-ΔUL21/US3Δkin	28	calm	Mild meningoencephalitis with single cell necrosis in Pir and LEnt, meningeal and perivascular infiltrates of T-lymphocytes and histiocytes as well as glial activation in the temporal lobe (Pir, Hpc, LEnt) and prefrontal cortex (AI)	IHC	CD3	0	2	2	2	2	1	0	1	0
						Iba1	-	+	+	-	+	-	+	-	+
						GFAP	-	+	+	-	+	-	+	-	-
						Cas3	0	0	1	0	1	0	0	0	0
					ISH	LLT	1	1	1	0	0	1	1	0	1
						UL19	1	1	1	1	1	1	1	1	0
M5	PrV-ΔUL21/US3Δkin	28	calm	Mild meningoencephalitis with single cell necrosis in Pir and LEnt, meningeal and perivascular infiltrates of T-lymphocytes and histiocytes as well as glial activation in the temporal lobe (Pir, Hpc, LEnt) and prefrontal cortex (AI)	IHC	CD3	1	3	3	3	3	0	1	1	0
						Iba1	+	-	+	+	+	-	+	-	-
						GFAP	-	-	+	+	+	-	-	-	-
					ISH	LLT	1	1	1	1	1	0	0	0	0
						UL19	1	2	2	3	2	2	0	1	0
M6	PrV-ΔUL21/US3Δkin	28	-	Mild meningoencephalitis with infiltrates of T-lymphocytes in the temporal lobe (Pir, Hpc)	IHC	CD3	0	1	2	2	1	0	1	0	0
						Iba1	-	-	-	-	-	-	-	-	-
						GFAP	+	-	-	-	-	-	+	-	-
					ISH	LLT	1	0	1	0	0	1	1	0	1

						UL19	0	1	1	2	1	1	1	1	1
M7	PrV-∆UL21/US3∆kin	42	-	Mild meningoencephalitis with single cell necrosis in Pir and Hpc, meningeal and perivascular infiltrates of T-lymphocytes and histiocytes as well as glial activation in the temporal lobe (Pir, Hpc, LEnt), parietal lobe (S1) and prefrontal cortex (AI)	IHC	CD3	0	2	3	2	2	2	1	1	0
						Iba1	-	+	+	-	+	+	+	-	-
						GFAP	-	+	+	+	+	+	+	-	-
						Cas3	0	0	1	1	1	0	0	1	0
					ISH	LLT	1	2	2	3	1	2	4	0	1
						UL19	0	1	1	1	1	1	1	1	0
M8	PrV-∆UL21/US3∆kin	42	-	Moderate meningoencephalitis with single cell necrosis in Pir, meningeal and perivascular infiltrates of T-lymphocytes and histiocytes as well as glial activation in the temporal lobe (Pir, Hpc, LEnt), parietal lobe (S1), prefrontal cortex (AI) and Cb	IHC	CD3	0	3	3	3	3	2	1	1	2
						Iba1	-	-	+	+	+	+	+	-	+
						GFAP	-	+	+	+	+	+	+	-	+
						Cas3	0	0	2	1	3	1	0	0	0
					ISH	LLT	0	1	1	1	1	1	1	0	0
						UL19	1	5	5	4	4	5	4	1	1
M9	PrV-∆UL21/US3∆kin	42	'star gazing'	Moderate meningoencephalitis with single cell necrosis in Pir, LEnt and neuron loss in Hpc, meningeal and perivascular infiltrates of T-lymphocytes and histiocytes as well as glial activation in the temporal lobe (Pir, Hpc, LEnt)	IHC	CD3	1	2	3	3	3	2	1	1	1
						Iba1	-	-	+	+	+	-	+	-	+
						GFAP	-	-	+	+	+	-	+	-	-
						Cas3	0	1	3	3	3	0	0	0	0
					ISH	LLT	0	1	1	1	1	1	1	0	1
						UL19	1	2	3	2	2	2	2	1	1
M10	PrV-∆UL21/US3∆kin	105	-	Mild meningoencephalitis with single cell necrosis in Pir and LEnt, meningeal and perivascular infiltrates of T-lymphocytes and histiocytes as well as glial activation in the temporal lobe (Pir, Hpc, LEnt)	IHC	CD3	0	1	3	2	3	2	1	n/a	1
						Iba1	-	+	+	+	+	-	-	n/a	-
						GFAP	-	-	+	+	+	-	-	n/a	-
					ISH	LLT	1	1	1	1	1	1	4	n/a	0
						UL19	1	3	4	3	3	4	3	n/a	1
M11	PrV-∆UL21/US3∆kin	105	back hair loss	Mild meningoencephalitis with single cell necrosis in Pir and LEnt, meningeal and perivascular infiltrates of T-lymphocytes and histiocytes as well as glial activation in the temporal lobe (Pir, LEnt), prefrontal cortex (AI) and Sp5	IHC	CD3	1	2	3	1	3	2	2	n/a	1
						Iba1	-	+	+	-	+	-	+	n/a	-
						GFAP	-	+	+	-	+	-	-	n/a	-
					ISH	LLT	1	2	2	1	1	1	4	n/a	1
						UL19	1	1	2	2	3	3	1	n/a	0
M12	PrV-∆UL21/US3∆kin	105	-	Mild meningoencephalitis with single cell necrosis in Pir and Hpc, meningeal and perivascular infiltrates of T-lymphocytes and histiocytes as well as glial activation in the	IHC	CD3	0	0	3	2	3	3	1	n/a	0
						Iba1	-	-	+	+	-	+	+	n/a	-

				temporal lobe (Pir, Hpc, Lent) and parietal lobe (S1)		GFAP	-	-	+	+	-	+	-	n/a	-
						Cas3	0	0	1	1	0	0	0	0	1
					ISH	LLT	0	1	1	2	1	1	4	n/a	0
						UL19	1	3	3	4	4	3	4	n/a	1
M13	PrV-ΔUL21/US3Δkin+ 170dpi cyclophosphamide/ dexamethasone treatment	190	-	Mild meningoencephalitis with single cell necrosis in Hpc, meningeal and perivascular infiltrates of T-lymphocytes and histiocytes as well as glial activation in the temporal lobe (Pir, Hpc, Lent) and parietal lobe (S1) and OB	IHC	CD3	2	1	2	3	2	2	1	1	0
						Iba1	+	-	+	+	+	+	-	-	-
						GFAP	-	-	+	+	+	-	-	-	-
					ISH	LLT	1	0	1	1	1	1	1	0	0
						UL19	0	0	1	1	0	1	0	1	0
M14	PrV-ΔUL21/US3Δkin+ 170dpi cyclophospahmide/ dexamethasone treatment	190	-	Mild meningoencephalitis with meningeal and perivascular infiltrates of T-lymphocytes and histiocytes as well as glial activation in the temporal lobe (Pir, Hpc) and prefrontal cortex (AI)	IHC	CD3	1	2	2	3	1	1	1	1	0
						Iba1	-	+	+	+	-	+	+	-	-
						GFAP	-	-	-	+	+	+	-	-	-
						Cas3	0	0	3	2	2	0	0	0	0
					ISH	LLT	1	1	1	1	1	1	1	0	0
						UL19	1	1	2	2	2	1	0	1	0
M15	PrV-ΔUL21/US3Δkin+ 170dpi cyclophospahmide/ dexamethasone treatment	190	calm	Mild meningoencephalitis with meningeal and perivascular infiltrates of T-lymphocytes and histiocytes as well as glial activation in the temporal lobe (Pir, LEnt) and OB	IHC	CD3	1	1	1	1	1	1	1	1	0
						Iba1	+	+	+	-	+	+	-	-	-
						GFAP	+	-	+	-	-	-	-	-	-
					ISH	LLT	2	1	2	2	1	2	1	0	1
						UL19	0	1	0	0	0	0	0	0	0
M16	mock + 170dpi cyclophospahmide/ dexamethasone treatment	190	-	-	IHC	CD3	0	0	0	0	0	0	0	0	0
						Iba1	-	-	-	-	-	-	-	-	-
						GFAP	-	-	-	-	-	-	-	-	-
						Cas3	0	0	3	1	1	0	0	0	0
					ISH	LLT	0	0	0	0	0	0	0	0	0
						UL19	0	0	0	0	0	0	0	0	0
M17	mock	21	-	-	IHC	CD3	0	0	0	0	0	0	0	0	0
						Iba1	-	-	-	-	-	-	-	-	-
						GFAP	-	-	-	-	-	-	-	-	-
						Cas3	0	0	0	0	0	0	0	0	0

					ISH	LLT	0	0	0	0	0	0	0	0	0
						UL19	0	0	0	0	0	0	0	0	0
