# Supplementary figures and images for: Regional and Temporal Patterns of Long-Term Pseudorabies Virus Detection and Neuropathology in the Murine CNS
Source: Pathogens. 2026 Apr 7;15(4):395. doi: 10.3390/pathogens15040395 (PMC13118813; doi:10.3390/pathogens15040395)

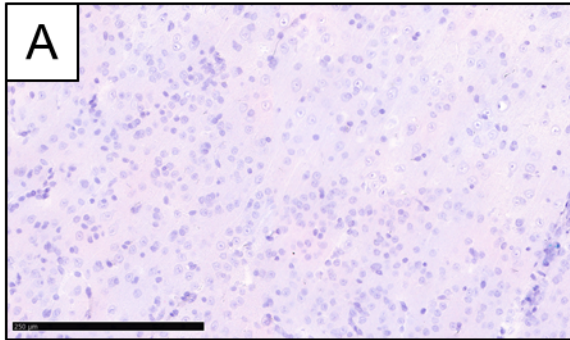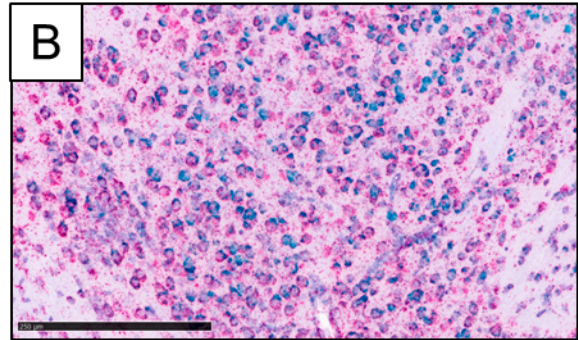

Supplement: Supplementary file 1 [file pathogens-15-00395-s001.zip › Figure S2.pdf]

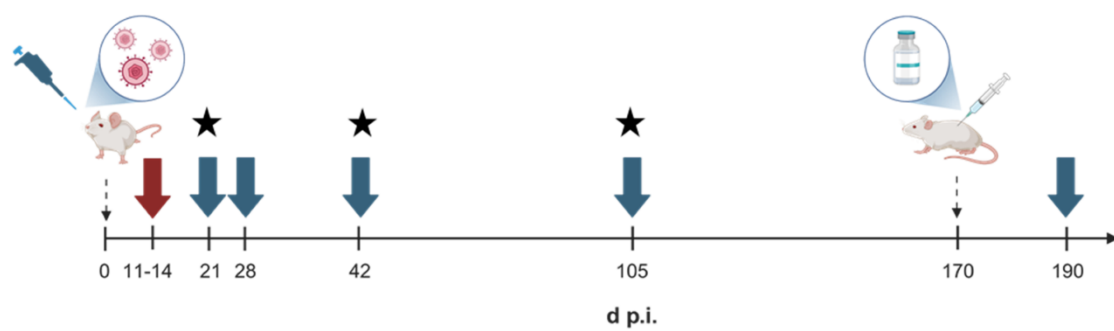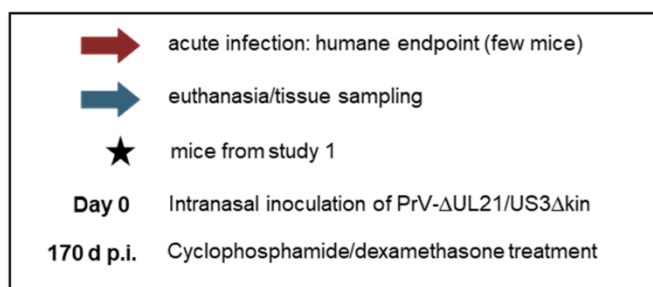

Supplement: Supplementary file 1 [file pathogens-15-00395-s001.zip › Figure S3.pdf]
